# Dynamics of Copy Number Variation in Host Races of the Pea Aphid

**DOI:** 10.1093/molbev/msu266

**Published:** 2014-09-18

**Authors:** Ludovic Duvaux, Quentin Geissmann, Karim Gharbi, Jing-Jiang Zhou, Julia Ferrari, Carole M. Smadja, Roger K. Butlin

**Affiliations:** ^1^Department of Animal and Plant Sciences, University of Sheffield, Sheffield, United Kingdom; ^2^Edinburgh Genomics, Ashworth Laboratories, University of Edinburgh, Edinburgh, United Kingdom; ^3^Department of Biological Chemistry and Crop Protection, Rothamsted Research, Harpenden, United Kingdom; ^4^Department of Biology, University of York, York, United Kingdom; ^5^Institut des Sciences de l’Evolution (UMR 5554), CNRS, IRD, Université Montpellier 2, Montpellier, France; ^6^Sven Lovén Centre for Marine Sciences–Tjärnö, University of Gothenburg, Strömstad, Sweden

**Keywords:** speciation, adaptation, targeted resequencing, gene duplication, gene family evolution, chemosensory genes, *P450*, *Acyrthosiphon pisum*

## Abstract

Copy number variation (CNV) makes a major contribution to overall genetic variation and is suspected to play an important role in adaptation. However, aside from a few model species, the extent of CNV in natural populations has seldom been investigated. Here, we report on CNV in the pea aphid *Acyrthosiphon pisum,* a powerful system for studying the genetic architecture of host-plant adaptation and speciation thanks to multiple host races forming a continuum of genetic divergence. Recent studies have highlighted the potential importance of chemosensory genes, including the gustatory and olfactory receptor gene families (*Gr* and *Or*, respectively), in the process of host race formation. We used targeted resequencing to achieve a very high depth of coverage, and thereby revealed the extent of CNV of 434 genes, including 150 chemosensory genes, in 104 individuals distributed across eight host races of the pea aphid. We found that CNV was widespread in our global sample, with a significantly higher occurrence in multigene families, especially in *Ors*. We also observed a decrease in the gene probability of being completely duplicated or deleted (CDD) with increase in coding sequence length. Genes with CDD variants were usually more polymorphic for copy number, especially in the *P450* gene family where toxin resistance may be related to gene dosage. We found that *Gr* were overrepresented among genes discriminating host races, as were CDD genes and pseudogenes. Our observations shed new light on CNV dynamics and are consistent with CNV playing a role in both local adaptation and speciation.

## Introduction

Within populations or among species, copy number variation (CNV) is a component of genetic diversity due to structural variation in genome content (Alkan et al. 2011) in which individuals harbor different copy numbers (CNs) of the same DNA sequence (the different copies being more or less similar depending on their history). CNV appears through duplication and deletion, the fate of new variants being driven by drift or selection, eventually contributing to the creation of genetic novelty (Lynch 2007; Innan and Kondrashov 2010). Understanding how genetic diversity contributes to adaptation and speciation is a crucial question in evolution (Wolf et al. 2010; Radwan and Babik 2012). CNV may play an important part: It has been shown to contribute significantly to genetic diversity in the wild (Feulner et al. 2013; Zichner et al. 2013) and to have important fitness effects on individuals, whether negative (e.g., in human diseases: Zhang et al. 2009) or positive (e.g., starch digestion: Perry et al. 2007; resistance to poison and pathogens: Labbé et al. 2007; Puinean et al. 2010; Eastman et al. 2011; Cook et al. 2012). Moreover, it is well known that many functionally important parts of the genome, including large multigene families, evolve extensively through neo/subfunctionalization of duplicated genes undergoing divergent or balancing selection. Examples include the MHC system (Traherne 2008), self-incompatibility systems in flowers (Charlesworth et al. 2005), and chemosensory genes (CSG) in mammals and insects (Cooper et al. 2007; Smadja et al. 2009; Briscoe et al. 2013; Karn 2013). However, probably because CNV is more complicated to investigate than single nucleotide polymorphism (SNP) and expression variability, its potential role in adaptive evolution of wild populations and in speciation has been relatively understudied to date, especially at the genome scale (Stapley et al. 2010; Ekblom and Galindo 2011; Seehausen et al. 2014, but see Feulner et al. 2013; Briscoe et al. 2013; Zichner et al. 2013 for recent CNV studies).

One of the most useful systems for analyzing factors, including CNV, that influence speciation is the formation of host races in phytophagous insects. Host races are populations in partial reproductive isolation that show local adaptation and preference for alternative habitats (host plants) whose distributions can be clearly defined (Drès and Mallet 2002; Bush and Butlin 2004; Matsubayashi et al. 2010). An excellent example of a host race complex is the pea aphid. It has been shown to form genetically distinct host-associated populations on multiple legume species that are considered to be host races or biotypes (Via 1991; Frantz et al. 2006; Peccoud, Ollivier, et al. 2009; Ferrari et al. 2012). Races have higher preference for and performance on the plant species that they have been found on in the wild compared to alternative host-plants (Via et al. 2000; Ferrari et al. 2008; Peccoud, Ollivier, et al. 2009). This host-plant specialization is the key component of reduced gene flow between races (Via 1999; Ferrari et al. 2006, 2012; Frantz et al. 2006; Peccoud, Ollivier, et al. 2009). Indeed, it is thought to induce selection against migrants from other host plants and against hybrids (Via et al. 2000), as well as assortative mating as pea aphids are thought to feed and mate on the same host species throughout their life cycle (Via 1999). A key feature which makes the pea aphid example a highly valuable host race system is the ability to study multiple levels of divergence within the same complex. In Europe, the races represent a continuum of differentiation from very low levels of genetic divergence, with ongoing gene exchange, to much higher divergence and more complete reproductive isolation (Peccoud, Ollivier, et al. 2009). This suggests that host races constitute an intermediate step in the speciation process, and that host specialization may indeed lead to complete speciation. The pea aphid is therefore ideal for comparing genomic patterns of divergence at different points along the speciation continuum, which can help in reconstructing how genomic divergence unfolds during speciation (Seehausen et al. 2014).

A key stage of host race formation is divergence between the race-specific sensory systems required to identify host plants. Although vision can help phytophagous insects to detect host plants from afar (Reeves 2011), they usually recognize their hosts thanks to chemical cues, this recognition involving CSG (Caillaud and Via 2000; Smadja and Butlin 2009). Aphids most likely assess their compatibility with plants utilizing volatile (Webster et al. 2008; Webster 2012) and, especially, nonvolatile chemicals during probing of subepidermal tissues (Pickett et al. 1992; Caillaud and Via 2000; Schwarzkopf et al. 2013). Although their putative function has yet to be properly confirmed in many cases, gustatory receptors (*Gr*) and olfactory receptors (*Or*) are strong candidates to play a role during this process. Genome studies of several *Drosophila* species (McBride and Arguello 2007; Gardiner et al. 2008a), the honey bee (Robertson and Wanner 2006), mosquitoes (Kent et al. 2008), moths and butterflies (Zhou et al. 2009; Briscoe et al. 2013), and *Tribolium castaneum* (Abdel-Latief 2007) have shown that CSG usually form very large multigene families and that their composition may strongly differ among species, even where closely related (Briscoe et al. 2013). Importantly, CSG evolve under a birth-and-death process, that is, successive duplications, deletions and pseudogenization events, with some recently duplicated genes potentially evolving under positive selection (McBride and Arguello 2007; Smadja et al. 2009). This extensive interspecific variation seems to be mirrored at the population level, although the picture is very incomplete so far (Briscoe et al. 2013). Even though CNV may often evolve neutrally (Craddock et al. 2010), selection is thought to be important to explain the evolution of large multigene families and several theoretical models support this view (Innan and Kondrashov 2010). In the pea aphid lineage, Smadja et al. (2009) detected a rapid expansion of the *Or* and *Gr* families with positive selection in some subfamilies. A recent outlier scan exploring patterns of differentiation at about 150 CSG among three aphid host races specialized on *Trifolium pratense*, *Medicago sativa*, and *Lotus pedunculatus* plants revealed 18 *Gr* and *Or* genes as highly divergent loci (Smadja et al. 2012), suggesting that they could play key roles in host-plant specialization. Using a microsatellite-based genome scan, Jaquiéry et al. (2012) also revealed a few *Or* in genomic regions strongly divergent between aphid host races. Altogether, these features make CSG very strong candidates to influence speciation in host-specialized insects. However, the role of CNV at these candidate loci in influencing adaptation to hosts, and hence speciation, remains to be tested.

In this study, we assessed the extent of CNV within and among eight host races of the pea aphid at about 500 CSG and control genes. We achieved very deep sequencing through targeted sequence capture (average 385X per target per individual) and we applied a new statistical framework (Rigaill et al. 2012), for the first time with population data. We investigated whether CNV in general, and CNV of CSG in particular, may have played an important role in divergence among pea aphid host races by testing for race-specific CNV patterns. We found that 1) multigene families, especially *Or* genes, showed more CNV than control genes; 2) coding sequence length (CSL) was negatively correlated with a gene’s probability of being completely duplicated or deleted (CDD); and 3) genes with CDD variants showed higher frequencies of variant copy numbers (CN ≠ 1) than genes without CDD variants, especially among the *P450* genes, a family known for its role in resistance to toxins. Finally, we observed that genes with CDD variants and *Gr* genes contribute strongly to discrimination among host races, reinforcing previous evidence (Smadja et al. 2012) that chemoreceptors, particularly members of the *Gr* family, play a key role in aphid host race formation.

## Results

### Capture Sequencing, Data, and Basic Statistics of CNV

#### Capture Sequencing Results

We designed a capture experiment including 3,343 unique targets from a set of 486 CSG and control genes (see Materials and Methods and supplementary tables S1 and S2, Supplementary Material online), using Assembly 1.0 of the pea aphid genome (International Aphid Genomics Consortium 2010). We sequenced 120 parthenogenetic aphid female lineages (hereafter clones) from eight races (Materials and Methods and supplementary table S3, Supplementary Material online). The Illumina sequencing generated a total of 3.1 billion paired-end reads resulting in a median target sequencing depth of 385X (the median over targets per aphid clone ranged from 98 to 1,330X; supplementary table S4, Supplementary Material online). The median target enrichment per clone varied between 34- and 214-fold. Capture metrics are provided in the supplementary figure S1, Supplementary Material online.

#### Data for CNV Estimation

We used the method of Rigaill et al. (2012) to estimate CNV by comparing the proportion of reads per base pair (PRbp) from any focal individual to a “gold standard” (i.e., the average PRbp of ten *Medicago* clones, see Materials and Methods). To account for differences in enrichment between libraries, the data were normalized using a polynomial transformation. After normalization, 104 clones were retained for subsequent analyses (supplementary table S3, Supplementary Material online). Accurate localization of CNV break points (i.e., start and end positions of structural variants) was obtained by subdividing targets into nonoverlapping “subtargets” of 50 bp for which CNV was estimated (see details in Materials and Methods). Because duplication of loci in the reference genome is known to influence CNV estimation (Teo et al. 2012), we focused our analyses on subtargets that were present at a single location in Assembly 2.1 of the pea aphid reference genome (see Materials and Methods and supplementary table S5, Supplementary Material online). After cleaning, the resulting data set contained 2,778 targets and 10,879 subtargets, representing about two-thirds of the initial targeted sequence length (supplementary table S2, Supplementary Material online).

#### CNV Was Widespread

**Fig. 1.**
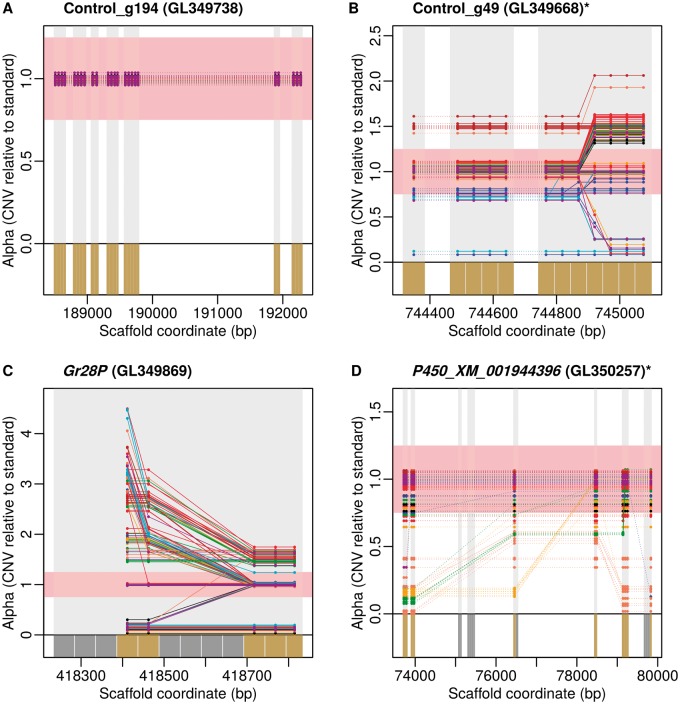
Examples of CNV estimation showing typical patterns. (*A*) Gene without CNV. (*B–D*) Genes showing both complete and incomplete duplications and/or deletions. Each line represents an individual (one color per race plus *Medicago* standard in purple). For clarity, values of alpha represented here are those before rounding to the closest half unit (the red box represents the area in which alpha values were rounded to 1). Vertical light gray-shaded areas represent targets as originally designed whereas bottom dark gray and gold boxes represent subtargets excluded and retained for final analyses, respectively. Retained subtargets are linked by full lines when from the same target and by dotted lines where not. Gene names (and scaffold numbers) are indicated above each plot. Control genes are shown with their alias names. The “P” in *Gr28P* stands for pseudogene. The “*” symbol indicates genes partially represented due to the absence of targets upstream or downstream filtered out during cleaning steps.

**Fig. 2.**
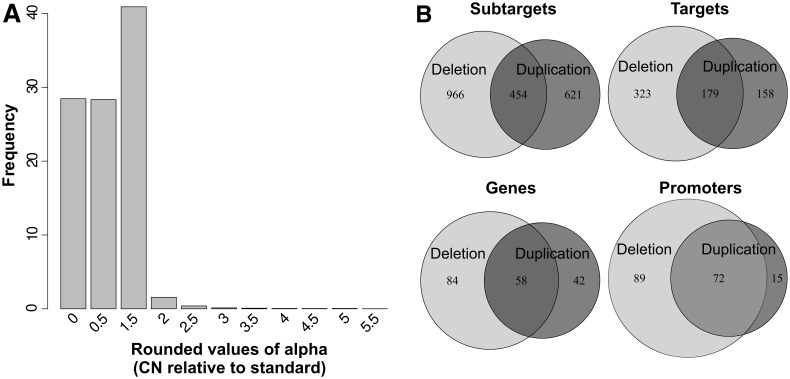
Distribution of CNV in the global sample. (*A*) Frequency distribution of CN variants across the global sample, excluding observations with CN = 1. One observation corresponds to one subtarget for one individual and CNs are expressed in proportion to the gold standard (i.e., rounded values of alpha). The sum of 1.5X < CN < 3X and 2.5X < CN represents about 2% and 0.35% of the observations, respectively. Subtargets with a CN = 1 are not shown but represent 98.09% of the total data set. (B) Partition of duplications and deletions by categories. One observation corresponds to one locus of the category represented.

A striking result was that CNV appears to be widespread in the pea aphid genome: 2,041 subtargets were polymorphic for CN in the global sample (18.8%), among which 31% were singletons (CN polymorphism present only once in the sample of 104 clones retained for analysis; fig. 1 and supplementary fig. S1, Supplementary Material online). This represents 24% of the targets distributed in 42% of the genes and 40% of the promoters (table 1). It is remarkable that as many as 30% of the randomly chosen control genes (see Materials and Methods) showed CNV even though they were selected to represent the whole genome, in contrast to multigene families for which we expected different CNV dynamics. Nevertheless, beside the small *Sp* (salivary protein) gene family, the gene families with the highest proportions of CNV were the three biggest multigene families in our data set (*Or*, *Gr* and *P450* with 64%, 61% and 56% of genes with CNV, respectively; table 1). Another interesting aspect is that, despite their very small length (≈50 bp, length of the output of the predictor program), making them a priori less likely to be included in a structural variant, loci predicted to be promoters were almost as variable in CN as genes (40% and 42%, respectively). Estimated CN ranged from 0 to 5.5 times the average number of copies of our gold standard (corresponding to alpha values on fig. 1). We observed many more values of 0X, 0.5X, and 1.5X than values of 2X or above (fig. 2*A*). Half values (0.5X, 1.5X, etc.) may commonly represent heterozygotes for deletions or duplicates. However, in some cases these values were consistent across individuals within one or more races, which is most likely to reflect an absolute CN ≠ 1 in the gold standard (a large excess of CN heterozygosity in populations being much less likely). Overall, deletions were more frequent than duplications (note that duplications and deletions were defined relative to the gold standard, unlike the evolutionary definition characterizing a derived mutation, therefore alpha values <1 and >1 were always considered deletions and insertions, respectively). This is to be expected from read mapping CNV detection methods (see Teo et al. 2012 and Discussion section). Interestingly, many targets presented both deletions and duplications in the global sample (individuals from all races included; fig. 2) or even within races (not shown).

**Table 1.** Distribution of CNV among Categories of Targets or Genes.

	Number of Loci	Polymorphic for CN	Proportion
Unit category			
Analysis units			
Subtargets	10,879	2,041	0.188
Targets	2,778	660	0.238
Functional units			
Genes	434	184	0.424
Promoters	441	176	0.399
Gene category			
CSG			
*Or*	67	43	0.642
*Gr*	62	38	0.613
*Obp*	11	2	0.182
*Csp*	10	0	0
Other multigene families			
*Ir*	11	3	0.273
*Snmp*	9	0	0
*P450*	60	34	0.567
*Sp*	5	4	0.8
*Ps*	5	0	0
Other			
Control	194	58	0.299
CSG promoters	441	176	0.399

Note.—Polymorphic loci can present deletions, duplications, or both.

### Dynamics of CNV

In order to understand the dynamics of CNV during speciation, we estimated the influence of several variables (including race effects) on CNV occurrence using generalized linear mixed models (GLMM). We restrained our analyses to *Or*, *Gr*, *P450,* and control genes as other categories were either not directly comparable (i.e., promoters) or had too few genes to allow proper statistical analyses. Thus, CNV was assessed for 381 genes across the eight races for a total of 3,048 observations.

#### *Or* Genes Were more Likely to Be Variable for CN

**Fig. 3.**
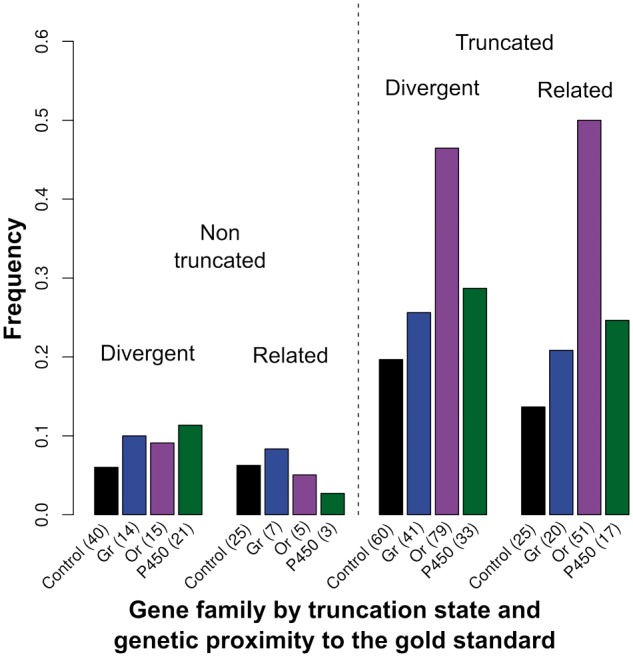
Proportion of observations with CNV per gene family, with races categorized by genetic relatedness to the gold standard. The races related to and divergent from the standard are *Medicago, Pisum, Trifolium* and *Lathyrus, Cytisus, Ononis, Lotus pedunculatus, Lotus corniculatus,* respectively (see Explanatory Variables Used in GLMM). The number of observations polymorphic for CN per category is indicated in parentheses. The total numbers of polymorphic observations were 150, 82, 150 and 74 for Control, *Gr*, *Or* and *P450*, respectively. These observations are distinguished between nontruncated genes (left side) and truncated genes (right side).

We started by investigating variables influencing the probability that a gene showed CNV (scored as present or absent, per race), regardless of whether CNV involved the complete sequence or not (i.e., partial or complete duplication/deletion; table 2 and fig. 3). Genes truncated during cleaning steps were shown to be more prone to CNV even though their length had been shortened artificially (logit estimate = 2.08, *P* < 0.001; table 2). This result is not surprising as genes were truncated precisely because part of their sequence was found to be duplicated in the reference pea aphid genome (see Materials and Methods). The fact that they are duplicated in the reference genome makes them more likely to be copy-number variable in natural populations. A more biologically significant feature is that *Or* genes were significantly more prone to CNV than control genes (logit estimate = 1.70, *P* < 0.001—note that all family comparisons were made using treatment contrasts against control genes as the reference group, see Materials and Methods). The same was true for *Gr* and *P450* genes although the differences were smaller (logit estimates 0.88 and 0.97, respectively; *P* < 0.05). None of the other variables had a significant effect on CNV probability although there was a tendency to detect less CNV in races closely related to the gold standard than in divergent races (fig. 3). The absence of an effect of either the coding sequence (CSL) or the intronic sequence length (ISL) is surprising as one might have expected the probability of being included in a structural variant to increase with gene length.

**Table 2.** Estimated Variable Effects (logit scale) from the GLMM on the Probability of CNV.

	Estimate	SE	*z* Value	Pr(>|*z*|)
Intercept	−4.669	1.585	2.945	0.003
CSL	0.137	0.414	0.331	0.741
ISL	−0.032	0.085	0.376	0.707
Truncated	2.077	0.291	7.134	<0.001
Family				
*Gr*	0.879	0.407	2.159	0.031
*Or*	1.692	0.393	4.305	<0.001
*P450*	0.974	0.411	2.369	0.018
GPS	−0.352	0.220	1.601	0.109
*Gr* × GPS^a^	−0.010	0.394	0.024	0.981
*OR* × GPS^a^	0.335	0.366	0.917	0.359
*P450* × GPS^a^	−0.525	0.427	1.23	0.219

Note.—GLMM was run for 8 races and 381 genes that are 3,048 observations. GPS, genetic proximity to gold standard.

^a^Interaction terms.

#### CSL Negatively Influenced the Probability of Complete Duplication/Deletion

**Fig. 4.**
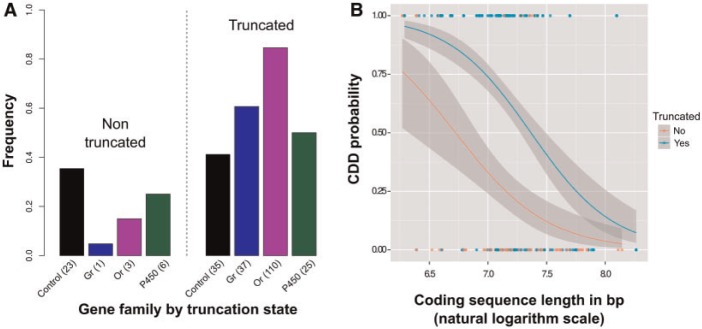
Proportion of genes polymorphic for CN that were CDD. (*A*) Proportion of CDD observations per gene family by categories of truncated genes. The number of CDD observations per category is indicated in parentheses. The total numbers of CDD observations were 58, 38, 113 and 50 for Control, *Gr*, *Or* and *P450* families, respectively. (*B*) Relationship between gene CSL and CDD probability.

We tested variables that could influence the probability that a gene was completely duplicated or deleted (CDD_j_: Genes showing CN ≠ 1 for all their subtargets in at least one individual of race j), given that CNV was present (456 observations). As expected, loci truncated during in silico cleaning steps were significantly more likely to be CDD (logit estimate = 3.89, *P* < 0.001; table 3 and fig. 4*A*). Although these genes might be more likely to be completely duplicated in natural populations, this pattern probably reflects the bias resulting from our cleaning steps, unlike the effect on CNV probability (see above), and so should be interpreted with caution. Interestingly, no significant difference was detected between control genes and multigene families. However, CDD probability was strongly and negatively correlated with CSL (logit estimate = −5.5, *P* < 0.001; table 3 and fig. 4*B*) even though there was no relationship with total ISL (logit estimate = −0.09, *P* = 0.67).

**Table 3.** Estimated Variable Effects (logit scale) from the GLMM on the Probability that Genes with CNV Show Complete Duplication/Deletion.

	Estimate	SE	*z* Value	Pr(>|*z*|)
Intercept	36.215	8.625	4.199	<0.001
CSL	−5.518	1.223	4.511	<0.001
ISL	−0.094	0.221	0.426	0.670
Truncated	3.889	0.817	4.758	<0.001
Family				
*Gr*	−0.588	1.011	0.582	0.560
*Or*	1.501	0.924	1.624	0.104
*P450*	0.593	1.054	0.563	0.573

Note.—The GLMM was run from 456 observations (i.e., one observation per pair gene/race where variation in CN has been found).

#### Genes with CDD Variants Showed Higher Frequencies of CN Variants in Populations

**Fig. 5.**
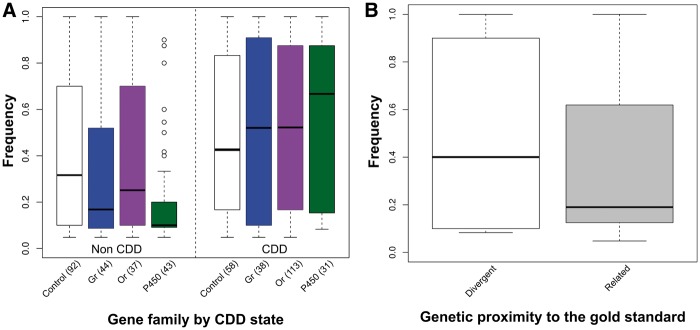
Frequency of CN ≠ 1 within races. (*A*) Frequency per gene family by category of CDD. (*B*) Frequency per category of genetic relatedness to the standard.

Finally, we tested variables potentially influencing the proportion of individuals with CN ≠ 1 (copy being either complete or partial), per race and gene, given that CNV was present (456 observations). The most significant effect was the positive influence of the presence of CDD variants (logit estimate = 1.20, *P* < 0.001; table 4 and fig. 5*A*). As truncated genes showed no significant difference in frequency from nontruncated genes (*P* = 0.14), the increase in frequency of individuals with CN ≠ 1 associated with CDD does not seem to result from our cleaning step. Because we considered all CN variants together, and because only one individual with a CDD variant was needed within a race for an observation to be considered CDD, the effect may have arisen because genes with higher frequencies of individuals with CN ≠ 1 were more likely to show CDD variants. Races closely related to the standard (logit estimate = −1.09, *P* = 0.016) showed lower frequencies of individuals with CN ≠ 1 (table 4 and fig. 5*B*). Note that, although the main effect of *P450* genes was not significant (*P* = 0.21), its interaction with CDD variants was significantly positive, meaning that CDD variants were associated with higher frequencies of individuals with CN ≠ 1 for *P450* than for control genes (fig. 5*A*, right panel).

**Table 4.** Estimated Variable Effects (logit scale) from the GLMM on the Frequency of CN Variants within Races.

	Estimate	SE	*z* Value	Pr(>|*z*|)
Intercept	−0.932	1.539	0.606	0.545
CSL	−0.135	0.369	0.366	0.714
CDD genes	1.205	0.331	3.644	<0.001
GPS	−1.091	0.453	2.408	0.016
Truncated	0.437	0.297	1.47	0.141
Family				
*Gr*	−0.200	0.469	0.427	0.670
*Or*	−0.148	0.457	0.323	0.747
*P450*	−0.806	0.648	1.244	0.214
CDD × *Gr*	0.871	0.701	1.242	0.214
CDD × *Or*	0.729	0.646	1.128	0.259
CDD × *P450*	1.941	0.770	2.521	0.012

Note.—The GLMM was run from 456 observations (i.e., one observation per pair gene/race where variation in CN has been found).

### Patterns of CNV Divergence among Races

**Fig. 6.**
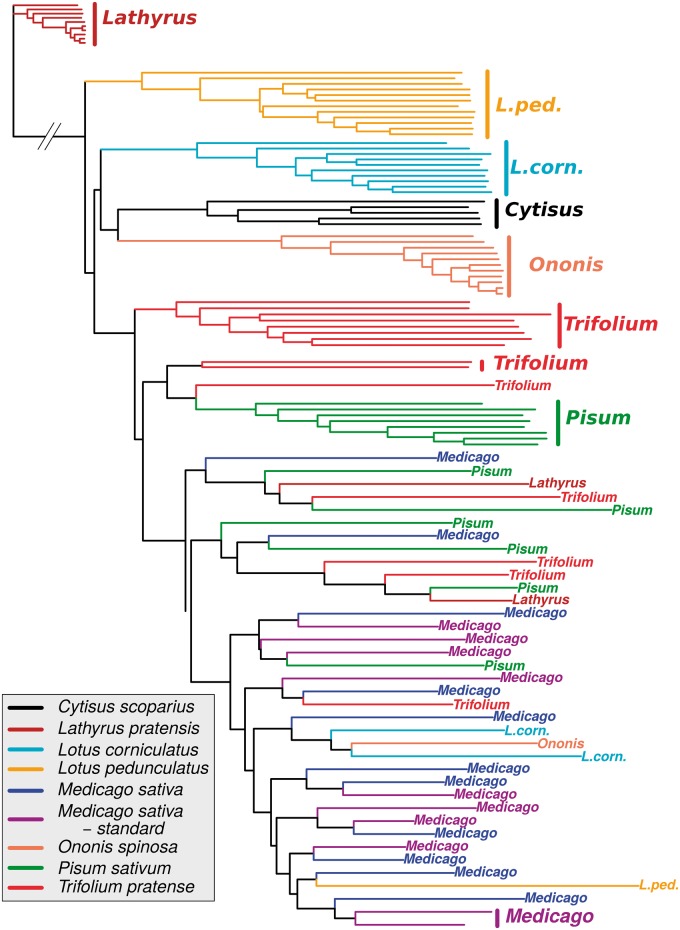
Neighbor-joining tree based on CNV information. The distance matrix was obtained by conversion of the RF proximity matrix computed from the 114 independent subtargets most informative to discriminate races. Clones from both the training and the test set are represented on the tree (see Materials and Methods). The branch leading to the *Lathyrus* outgroup has been shortened by a factor 4.6.

Given the evidence that the pattern of CNV differed among gene categories, we examined CNV patterns among races to test for a possible role in race divergence. We used the V_ST_ statistic, an analog of F_ST_ for CN (Redon et al. 2006), in order to describe the distribution of CNV within and among races. The among-race variation constituted 11–28% of the total variance in CN, with similar distributions in the four main categories of genes (median and interquartile range: Control 0.138 [−0.04, 0.308]; *Gr* 0.112 [0.009, 0.448]; *Or* 0.282 [0.135, 0.506]; *P450* 0.240 [0.045, 0.506]). However, this global V_ST_ analysis does not necessarily identify the loci that best discriminate among races. The best discriminating loci may, in some cases, be fixed differently between one or more pairs of races but have much shared variation in others. Relative to within-race variance in CN, this pattern would not lead to high variance among races overall, and would result in low to moderate values of V_ST_. Therefore, we used a recursive random forest (RF) analysis in order to estimate the importance of subtargets as discriminators among races based on CNV information alone. We chose to focus our RF analysis on subtargets and not genes because many individuals show partial duplication/deletion for many genes (fig. 1, Materials and Methods). Figure 6 shows the results of the RF classification based on a final set of 114 independent subtargets (list and importance estimates in supplementary table S6, Supplementary Material online), confirming that CNV was partly structured among races. We then tested whether any gene families were overrepresented among these subtargets. Of the 11 categories of loci investigated, only *Gr* were significantly overrepresented in the set of 114 subtargets (*P* value < 0.001; table 5). However, we observed a major drop in the RF information criterion for subtargets beyond the 40th position (supplementary fig. S2, Supplementary Material online) and therefore we repeated the test for the 40 best subtargets only (hereafter “top 40”). We still detected an overrepresentation of *Gr* (*P* = 0.007; table 5). Moreover, their ranks in the top 40 were higher than expected at random (*P* = 0.039; *P*>0.15 for all other categories). We also observed a high number of *Gr* and *Or* promoters in the top 40 (supplementary table S6, Supplementary Material online) although this pattern could not be tested statistically for each family independently. Another interesting aspect is that the CN differentiation of many genes seems to have been associated with a loss of function: Two of six *Gr* and five of five *Or* present in the top 40 may be pseudogenes, according to the analyses of Smadja et al. (2009). Note, however, that errors in the reference genome could have led these authors to miss-identify fully functional loci as pseudogenes. Moreover, for each of the subtargets in the final set, we determined which race was best discriminated (supplementary table S6, Supplementary Material online). Out of the top 40 subtargets, 25 showed deletions in the race they discriminated most strongly (i.e., CN < 1X). Interestingly, for genes duplicated in the best discriminated race (by RF importance) 14 appear in the top 40 of 17 present in the top 114. For each of the genes in the final set of 114 subtargets (promoters excluded), we also checked whether the gene showed CDD in the best discriminated race. We detected 17 CDD genes, all included in the top 40. Interestingly, they had higher ranks than expected at random (*P* < 0.001, see supplementary table S6, Supplementary Material online, for the list).

**Table 5.** Overrepresentation of Gene Families among the Most Informative Subtargets for Distinguishing Races Based on CNV Information.

	Top 114		Top 40	
	Head Count	*P* Value^a^	Head Count	*P* Value^a^
*Snmp*	1	0.556	1	0.071
*Control*	36	0.890	10	0.461
*Csp*	0	0.682	0	0.361
*Gr*	14	<0.001	6	0.007
*Ir*	1	0.701	0	0.454
*Obp*	0	0.852	0	0.417
*Or*	13	0.180	5	0.104
*P450*	15	0.110	4	0.198
Promoters	32	0.584	14	0.925
*Ps*	1	0.272	0	0.230
*Sp*	1	0.286	0	0.243

^a^One-tailed probability that the null hypothesis of no overrepresentation is true.

## Discussion

In this study, we used next generation sequencing (NGS) to investigate the dynamics of CNV in eight host races of the pea aphid, a phytophagous insect of special importance for the study of local adaptation and speciation. We took advantage of capture sequencing and recent bioinformatics developments to focus with great sensitivity on chemosensory and other multigene families that are candidates for a role in pea aphid host race formation. We discuss 1) advantages and limitations of our approach to investigate CNV, 2) the insights provided by our study concerning the extent of CNV in natural populations, 3) what our results suggest about the dynamics of CNV in the pea aphid, and 4) the role of CNV and, specifically, CSG families in host race formation in the pea aphid.

### A New Approach to Detect CNV in Natural Populations

#### Limitations of Current Approaches to Detect CNV in Natural Populations

Until recently, detection of CNV was restricted by methodological limitations. On the one hand, the analysis of small samples of complete genomes allowed the comparison of multigene families among divergent lineages, focusing on gene copies that have already been fixed over relatively long periods of time (Robertson et al. 2003; McBride and Arguello 2007; Gardiner et al. 2008a; Smadja et al. 2009). On the other hand, the most common way to detect and genotype CNV in larger intraspecific samples was to perform Sanger sequencing of paired reads followed by quantitative polymerase chain reaction (qPCR) characterization of further individuals (Dempsey et al. 2006; Teo et al. 2012). The recent development of SNP and comparative genomic hybridization arrays (Alkan et al. 2011) allowed more systematic characterizations of larger samples of loci and individuals simultaneously, but at high cost. Therefore, there remains an urgent need to study CNV more widely in the population context of adaptation and speciation. The availability of NGS has made it possible to detect and genotype CNV for many individuals at a genomic scale (Mills et al. 2011; Briscoe et al. 2013; Feulner et al. 2013; Zichner et al. 2013). However, most current methods rely on whole-genome sequencing (WGS) which has two main disadvantages. First, WGS is still prohibitively expensive for large scale population studies or has to be restricted to organisms with small genomes (a good example of this constraint can be seen in Feulner et al. 2013). Second, the relatively shallow sequencing performed increases the rate of false positives and negatives, notably for duplications relative to the reference genome (Teo et al. 2012). Here, we developed a pipeline to extend NGS approaches using capture sequencing which makes possible the high read-depth needed for accurate characterization of CNV content while still covering many loci (2,778 target regions) and many individuals (104 from eight races of the pea aphid). Finally, the small size of our subtargets (50 bp) allowed us to obtain good resolution of the breakpoint locations, which is important for functional analyses and mechanistic studies of CNV formation.

#### Potential Limitations of Our Approach to Detect CNV

One limitation of our approach is that the CNV estimates obtained are ratios of the number of copies in the focal individual to the average CN of the individuals of the gold standard. Therefore, our CN estimates do not reflect the absolute number of copies for loci where CN ≠ 1 in the gold standard (which may be true even where one copy is present in the reference genome) and may be inaccurate for loci showing high variance in CN among the gold standard clones. Indeed, our data suggest that the absolute number of copies in the gold standard was not actually 1 in all cases (fig. 1). However, we do not expect this issue to have influenced our main interpretations. First, these cases are expected to be relatively uncommon. Second, our rounding to the closest half-unit (see Materials and Methods) tended to reduce the false discovery rate (FDR) for loci where the average absolute CN of the gold standard was between 0.8 and 1.33 copies per chromosome. Third, we never used the absolute number of gene copies in any of our analyses. Instead, we concentrated on whether genes were CN variable or not. Finally, our rounding might have led to some false negatives. We believe that this strategy was conservative as false negatives were most likely in the most variable gene families and would tend to reduce apparent CNV for these families preferentially. Although common to all CNV detection methods based on read mapping, two more limitations should be noted: The positions of the new copies in the genomes cannot be determined and CNV could not be detected for loci absent in the reference genome. Again, we do not expect these issues to have influenced our main results.

### Extent of CNV in Natural Populations

In agreement with previous studies, we found that CNV is widespread along genomes and frequent in populations. Surprisingly, even randomly chosen control genes showed a high proportion of CNV (30% to be compared with the 7% and 6% of coding sequence reported in studies by Feulner et al. [2013] and Zichner et al. [2013], respectively). Part of this difference might be explained by a higher FDR of our method. Rigaill et al. (2012) report that only 70% of CNVs detected in their analysis were confirmed using comparative genomic hybridization arrays. Other methods (Pindel, CNVnator, or DELLY) (Abyzov et al. 2011; Rausch et al. 2012; Zichner et al. 2013) claim lower FDR but estimates are not directly comparable because different samples, data types, and reference standards were used. The normalization and rounding steps in our data analysis were conservative, if anything tending to underestimate the number of CNV loci. Note also that any indel as small as a subtarget (i.e., 50 bp) was considered as a CN variant in our study whereas the resolution is not always clear in other studies. Alternatively, our larger and more heterogeneous sample (104 individuals from eight genetically divergent host races) may have led us to detect more CNV genes. The high proportion of singletons (30%, value comparable to Feulner et al. [2013]) may also have increased the total number of genes with CNV. In pea aphid, this may reflect a recent population expansion, which has been suggested to have accompanied race formation (Peccoud, Simon, et al. 2009). Finally, it is worth noting that, with 35,000 genes (International Aphid Genomics Consortium 2010), the pea aphid genome has the largest gene content of all arthropods sequenced so far, indicating that it might be generally prone to CNV.

We detected an almost equal number of deletions and duplications, whereas previous studies have detected a large excess of deletions (typically 75–90%, see Teo et al. 2012; Feulner et al. 2013; Zichner et al. 2013). Indeed, methods to detect CNV are well known to detect duplications less efficiently than deletions (Alkan et al. 2011; Teo et al. 2012). Although it is difficult to compare results from such different studies, some factors may explain the greater proportion of duplications in our data. First, we limited ourselves to studying targets present in single copy in the reference genome (see Materials and Methods). This helps to overcome two problems (Teo et al. 2012): 1) Biases when using reads aligning multiple times on the reference genome, and 2) the increasing difficulty of detecting significant differences between *N* and *N* + 1 copies as *N* becomes larger (here, *N* refers to an absolute number of copies but the same reasoning can be applied to our alpha ratio). The second reason is that capture sequencing focused our analyses on a subset of genes only, allowing us to obtain extremely high depth of sequencing and thus to infer deletions and duplications reliably, even when CNs were large. Inefficient capture for some races does not seem to have been a problem as this would have falsely increased the proportions of deletions. The large number of duplications detected may have contributed to the fact that we observed a high overall proportion of CNV. Our approach, based on very high depth of sequencing, seems to be better able than other methods to detect duplications making it hard to determine whether they are actually more common in pea aphid than in other systems. Biologically, our results suggest that duplications could be almost as frequent as deletions in pea aphid populations, with many loci presenting both kinds of structural variant.

### Dynamics of CNV in the Pea Aphid Genome

It is expected that mutational mechanisms, genetic drift, and selection all influence the pattern of CNV observed within and among populations. Our data provide some insights into these processes.

#### 

##### Effect of the Mechanisms of CN Change on CNV Patterns.

Both sequence patterns within loci and the architecture of their immediate genomic neighborhood may influence the probability of CN change. For example, regions enriched in low CN repeats (e.g., nonallelic homologous exons from large multigene families) have increased rates of both homologous and nonhomologous recombination potentially generating CNV (Hastings et al. 2009). Note that nonallelic homologous exons can belong to one (Gardiner et al. 2008b) or several genes (Zichner et al. 2013) and that the presence of intronic transposable elements may have the same effect on nearby exons (Hastings et al. 2009; Janoušek et al. 2013). Thus, we can predict that genes found to be partially duplicated in the reference genome (“truncated genes” in table 2) and genes from families with recent and dynamic histories of duplication (i.e., including many genes with high sequence similarities) should present more CNV. As expected, “truncated genes” show more CNV than other genes and *Or* and *Gr* are more often truncated than other genes (31% for control genes and 53%, 51% and 38% for *Gr*, *Or*, and *P450* genes, respectively; generalized linear model *P* value for *Or* and *Gr* genes <0.01). Note, however, that assembly errors, for example, merging of several copies of an exon into one, may have artificially produced truncated genes and thus may have partly contributed to the observation that “truncated genes” show CNV more often. *Or* and *Gr* genes have a long history of duplication with many copies specific to the pea aphid lineage (Smadja et al. 2009). In line with our predictions, *Or* genes, which show the most recent and abundant duplication events (Smadja et al. 2009), also show the highest rate of CNV in this study (table 2; note that this pattern is also significant for *Gr* although less pronounced). Interestingly, Briscoe et al. (2013) recently showed that this prediction was also upheld for *Heliconius melpomene,* but for *Gr* genes only.

We detected a negative effect of the total CSL on CDD probability, whereas we observed no significant effect of total ISL (here used as a proxy of gene length; table 3). Can this apparent contradiction be explained in terms of mutational mechanisms? A key observation is that CSL was positively correlated with the number of exons in our data set (true for *Or*, *Gr* and control genes, Spearman rho: 0.66, 0.55, 0.77; all *P* values < 0.001). Exons are expected to lose sequence similarity with paralogs more slowly than introns. Long duplications/deletions commonly result from nonallelic homologous recombination (NAHR; Hastings et al. 2009). If exons represent islands of high sequence similarity within genes that can anchor NAHR, then the probability of partial duplication/deletion is expected to increase with exon number (and hence total CSL) but not with intron length. This would explain the correlation we observe. As recently duplicated exons are commonly involved in alternative splicing, these partial duplication/deletion events may have functional consequences (Letunic et al. 2002). If mechanisms other than NAHR (such as retrotransposition) commonly generate duplications/deletions, this could explain the contrast we observe between CDD probability, which is correlated with total CSL, and overall CNV probability, which is not (compare tables 2 and 3).

Generally, CNV is less common among closely related races than among more distant races (fig. 3, truncated genes), as expected from neutral accumulation of divergence. However, this is not the case for the *Or* gene family. This suggests a more rapid turnover of *Or* genes that could be due either to mutational effects or to selection.

##### The Effect of Selection.

Selection is expected to influence the pattern of CNV along genomes and variant frequency in populations (Innan and Kondrashov 2010). Either positive or purifying selection might influence the presence or the population frequency of either entire duplications/deletions or parts of them, following recombination. Here, we observed that genes with CDD variants had significantly higher frequencies of CN variants than other genes. One possible explanation is that incomplete duplicates are more likely to be kept at low frequency by purifying selection because they are more often mildly deleterious. Complete duplicates might also be more likely to reach high frequencies under positive selection. This might be the case for some *P450* genes that are known for their important detoxification roles, which may be dosage-dependent (Innan and Kondrashov 2010; Puinean et al. 2010). However, relaxation of purifying selection can also explain high frequencies of CN variants and may occur when genes are silenced. In our data set, some putative pseudogenes that show high frequency of CN variants within and among populations may be explained in this way (supplementary table S6, Supplementary Material online). We cannot exclude the possibility that undetected pseudogenes contribute to the pattern we observe for the *P450* family.

### The Role of CSG in Host Race Differentiation

Host races are thought to diverge by switching to new host plants, the switch involving loss of preference and/or adaptation to ancestral hosts as well as gain of new preferences and adaptations (McBride and Arguello 2007). As chemosensory receptors are probably involved in host preference in phytophagous insects (Smadja and Butlin 2009), they may contribute to the evolution of host races. Therefore, it is particularly interesting to find that *Gr* are overrepresented among subtargets that discriminate host races. Although they were not significantly overrepresented, we also observed many promoters of *Gr* and *Or* in the top 40 subtargets. This is consistent with the proposed functional importance of these families in host race differentiation and with evolution under divergent selection (Smadja and Butlin 2009). Other studies have also found that *Or* and *Gr* quickly evolved new distinct copies along diverging lineages (see fig. 10 in Briscoe et al. 2013). However, both McBride and Arguello (2007) and Briscoe et al. (2013) found that *Gr* were much more prone to pseudogenization and gene loss whereas *Or* tended to be more conserved among lineages, a trend that seems to be in the opposite direction for the pea aphid. Like these two studies, we detected more CN differentiation among lineages for *Gr* than for *Or*, but this difference was less pronounced in our study.

For both families, and their promoters, we found that most genes in our top 40 subtargets seem to have evolved following loss of function as they were either pseudogenes (mainly *Or*) or showed deletions in their sequences (mainly *Gr* and promoters). If nonfunctional copies are nearly neutral, and so spreading by drift, a high rate of CNV may cause *Gr* and *Or* to be overrepresented in the top 40 subtargets. Under this hypothesis, the most discriminating genes would be those that had had a long time to accumulate frequency differences among their CN variants. However, this hypothesis cannot explain all fixation events, especially those involving very short time scales, including cases where some lineages fix nonfunctional versions of genes that appear to remain functional in sister lineages (as witnessed by their low Ka/Ks in the latter). McBride and Arguello (2007) therefore proposed an interesting hypothesis to explain how specialist phytophagous insects could lose the functionality of some genes in the context of a host-plant shift. The idea is that the interactions of the insect with its new host and its associated pathogen community can render useless or even harmful some genes that were of great importance before. For example, bitter compounds in plants tend to deter feeding, indicating either toxins or pathogens. Phytophagous insects recognize them in order to identify a plant as unsuitable, this role being held by CSG. During a host switch, loss of function of such genes may be adaptive if they were previously used to recognize the new host as unsuitable. As an example, *Drosophila sechellia* is thought to have lost its repulsion response to the main toxin of *Morinda citrifolia* through the loss of expression of an odorant binding protein (*Obp*) gene in the gustatory hairs of its foretarsi (Matsuo et al. 2007). The same principle may apply for *Gr* in host races of the pea aphid as they are known to recognize their host plant based on metabolites detected during probing (Caillaud and Via 2000). Whether or not this process occurs in the pea aphid remains unknown. Del Campo et al.(2003) showed that, for at least two races of the pea aphid, the feeding behavior depends more on stimulants specific to the host plant rather than deterrents specific to the nonhost plant. Beside this appealing hypothesis, adaptive loss may also merely happen if a gene no longer required was costly, for example in terms of energy expenditure. In any case, these two possibilities probably apply to a minority of cases only, with loss of function more commonly being due to the relaxation of purifying selection on genes made nonessential after a host shift.

In a few cases, evolution of a new function may have led to fixation of a new variant in a given race. For example, Briscoe et al. (2013) showed that, in the butterfly *H. melpomene*, most of the *Gr* that are differentially expressed between males and females, and putatively involved in host-plant recognition for oviposition, were duplicated genes. Similarly, a newly duplicated *Gr* in any pea aphid host race might be involved in the recognition of its new host plant. Strong frequency differences among races for putatively functional CN variants were seen for *Gr4, Gr10,* and *Gr41*, for example.

Interestingly, the proportion of deletions was higher for *Gr* and *Or* than for other gene families (promoters excluded) in our top 40 (11/11 and 6/15, for *Gr* and *Or* together and for other genes, respectively; supplementary table S6, Supplementary Material online). This suggests that these other genes, particularly those showing CDD variants, may be more prone to evolve new functions that contribute to race formation. Given their functions in detoxification and resistance to toxins, and in synthesis of secondary metabolites, sex and alarm pheromones, the *P450* family genes are of special interest in this context (Schuler 2011). Indeed, all four *P450* genes present in the top 40 were CDD and three showed duplications, suggesting potential gene dosage effects. For instance, as hybrids have an intermediate numbers of copies, gene dosage effects in some *P450* genes could explain the intermediate performance of pea aphid hybrids (Peccoud et al. 2014). However, supplementary functional analysis, association studies, and/or expression data for the different variants within and among races will be needed to conclude definitively what drove the pattern of CNV observed in our study.

## Conclusion

Evidence is rapidly accumulating that structural variation, particularly in CN, is common within and among natural populations and that some of this variation has adaptive significance. Here, we have demonstrated that a targeted enrichment approach can allow sensitive detection of CNV across many loci and individuals, thus facilitating population-level analyses. Our large sample provides insights into the evolutionary dynamics of CNV. CNV is widespread in the pea aphid, particularly in the *Or* and *Gr* gene families. The gustatory receptor family shows greater than background levels of CN differentiation between host races of aphids and the odorant receptor family shows more CNV than other classes of genes in closely related races. There is clearly the possibility that CN evolution in these families has contributed to local adaptation and speciation.

## Materials and Methods

### Aphid Sampling, Rearing, DNA Extraction

Pea aphids (*Acyrthosiphon pisum* (Harris)) were collected from eight plant taxa that are known to host genetically distinct races (Peccoud, Ollivier, et al. 2009; Ferrari et al. 2012): *L. pedunculatus* Cav., *L. corniculatus* L., *M. sativa* L., *T. pratense* L., *Lathyrus pratensis* L., *Pisum sativum* L., *Cytisus scoparius* (L.) Link, and *Ononis* spp. (*O. spinosa* L. and *O. repens* L.). Because of parthenogenesis in pea aphids, the aphids were sampled from plants that were separated by at least 30 m to avoid collecting the same genotype multiple times. For each plant species, at least four geographically separate sites were used and the distance between these sites was always less than 100 km. The collections were made in south-east England in 2003, 2008, or 2010.

To produce sufficient DNA, the aphids were reared on broad bean (*Vicia faba* L.) in the laboratory. Cultures for each genotype were established from one field-collected parthenogenetic female. DNA was extracted from ten adults using the NucleoSpin Tissue kit (Macherey-Nagel GmbH & Co.) following the manufacturer’s instructions.

### Target Design

In order to enrich genomic DNA for genes of interest prior to sequencing, we used the SureSelect system (Agilent Technologies), which uses RNA probes (baits) designed to capture regions of interest from genomic DNA in solution (Gnirke et al. 2009; Mamanova et al. 2010).

Our main targets were a set of candidate genes potentially involved in the recognition of the host plant, including all of the *Or* genes (385 exons, 79 genes), *Gr* genes (358 exons, 77 genes), *Obp* genes (77 exons, 11 genes), and chemosensory protein (*Csp*) genes (21 exons, 10 genes) that had been partially or fully annotated in Assembly 1.0 of the pea aphid genome (Smadja et al. 2009; Zhou et al. 2010), as well as putative *cis*-regulatory sequences of all these genes. We also included other multigene families that may play a role in chemical signal reception or transduction, the ionotropic glutamate receptor (*Ir*) genes (Croset et al. 2010) (104 exons, 11 genes) and the sensory neuron membrane protein (*Snmp*) genes (71 exons, 9 genes), the *P450* gene family (493 exons, 69 genes) (Zhang et al. 2010) because of a potential role in detoxification of host-specific compounds, the pheromone synthesis proteins (*Ps*) (30 exons, 5 genes), which may contribute to reproductive isolation between races, and the *Sp* genes (28 exons, 5 genes), which may be involved in host-plant specific interactions during feeding. As controls, we randomly chose 211 genes (equivalent to 1,386 exons) in the list of annotated genes of the pea aphid genome that have a priori no known function related to host recognition and have gene family membership typical of the gene complement as a whole.

Primary sequence data for all target genes were extracted from Assembly 1.0 of the pea aphid genome using Apollo-AphidBase (International Aphid Genomics Consortium 2010). We searched putative *cis*-regulatory regions for each of the full-length *Obp*, *Csp*, *Or,* and *Gr* genes using the Neural Network Promoter Prediction program (http://www.fruitfly.org/seq_tools/promoter.html, last accessed September 24, 2014) with a score cutoff of 0.8. As a result, up to twenty 50-bp predicted promoter regions were added as targets for most of these genes. In addition, we included in the design 500-bp long sequences upstream of the start codon of all *Snmp* and *Ir* genes, which could potentially contain *cis*-regulatory regions. Overall, the initial capture target represented about 720 kb of sequence, including both candidate and noncandidate loci (supplementary tables S1 and S2, Supplementary Material online).

### Bait Design, Library Preparation, and Sequencing

The bait library was designed using the program eArrayXD, part of the Agilent Genomic Workbench 6.5 Standard Edition and Assembly 1.0 of the pea aphid genome as the reference genome (International Aphid Genomics Consortium 2010). We designed 120 base oligonucleotide baits with a tiling frequency of 4X (avoiding standard repeat masked regions but allowing a maximum overlap into avoided regions of 20 bp). The final design included 3,343 targets with baits, among the 3,610 initial target sequences we had selected (supplementary table S1, Supplementary Material online), and directly targeted 692 kb of sequence with 20,378 baits. Enriched libraries were prepared following Agilent’s SureSelect Target Enrichment System for Illumina Paired-End Sequencing Library protocol v1.2 with some modifications to accommodate precapture pooling of samples. Briefly, 3 µg of genomic DNA was sheared using a Covaris E210 focused ultrasonicator as per the manufacturer’s instructions. Following bead-based purification, sheared DNA was end-repaired, A-tailed, and adapter-ligated as recommended in the SureSelect protocol except that the Illumina PE Adapter Oligo Mix was used in the ligation reaction. Ligated libraries were enriched through six cycles of PCR following the SureSelect protocol using Illumina PE1.0 and custom index-specific PCR primers. Enriched libraries were quantified by picogreen and pooled in groups of six before capture. Hybrid capture selection and amplification of the library pools were carried out as recommended in the SureSelect protocol, replacing Indexing Block reagents by PE blocking reagents. Final capture pools were checked on a Bioanalyzer High Sensitivity DNA Chip (Agilent Technologies), quantified by qPCR (Kappa Library Quantification Kit), and sequenced at two libraries per lane of either Illumina GAIIx (v5 chemistry) or HiSeq 2000 (v1.5/v3 chemistry). Raw GAIIx and HiSeq reads were processed using RTA 1.9.35/Casava 1.7.0 and RTA 1.12.4.2/Casava 1.8.2, respectively.

### Processing of Sequencing Results

Sequence data were processed using a standard workflow (see, e.g., Feulner et al. 2013). Briefly, we obtained sequencing statistics using FastQC and then mapped reads on the Assembly 2.1 of the pea aphid genome using the most sensitive options of Stampy 1.0.17. Subsequent basic file manipulations including sam to bam file conversion, file merging, PCR duplicate removal, and alignment statistic computation were done using Samtools and Picard tools (http://picard.sourceforge.net/, last accessed September 24, 2014). SNP calling was performed for all 120 clones by using GATK 2.3-9 (DePristo et al. 2011) following a two-step procedure: 1) A local realignment around indels (RealignerTargetCreator and IndelRealigner tools) and 2) a one-pass SNP calling (UnifiedGenotyper tool).

### Clone Assignment to Host Race Using RF

Because hybrids or migrants from another host plant may be collected during sampling, clone membership to host races was confirmed by genotyping. For clone assignment analyses, we retained a set of 1,777 high-quality SNPs by removing SNPs: 1) Present in targets with CN ≠ 1, 2) with a Phred scaled probability (GATK “QUAL” criterion) <200, and 3) a proportion of heterozygotes >80% in the global sample. We applied an RF classification to the 120 individuals using these 1,777 SNPs (coded 0, 1, 2 according to the number of copies of the reference allele) using the “randomForest_4.6-7” package in R (Liaw and Wiener 2002; R Development Core Team 2013). Briefly, the RF methodology is a machine learning approach based on decision trees that can 1) define homogenous groups of similar individuals, 2) estimate variable importance for group discrimination, 3) run efficiently even using thousands of variables, 4) generate an internal unbiased estimate of the group discriminating error, 5) detect outliers, and 6) assign group probability to unknown samples (Breiman 2001; Touw et al. 2013; http://www.stat.berkeley.edu/∼breiman/RandomForests/cc_home.htm, last accessed September 24, 2014). First, we ran an “unsupervised” analysis (i.e., without prior assignment of clones to races, ntree = 5000, defaults for other parameters) and inspected the resulting grouping in relation to the host plant from which each clone was collected. Two clones from *Pisum* were found to be genetically identical (Pisum-121-T91 and Pisum-5-T100). Aphids from *Medicago* formed two genetically distinct clusters. These were kept separate in the following RF analyses but combined in the CNV analyses. Their status will be discussed in another study. We then selected a training set of 97 clones for which the grouping was concordant with the collection host and used this set in a second, supervised RF analysis (supplementary table S3, Supplementary Material online). This second analysis was refined by removing SNPs with low importance (Mean Gini Importance < 0.05). The final RF, using 511 informative SNPs, was used to classify the 22 clones not included in the training set. Clones were then assigned to races on the basis of their probabilities of group membership: Race assignments were made where the probability was greater than 0.45 for the most likely race, which had to correspond to the race of the collection plant, and lower than 0.25 for the next most likely race. Where these criteria were not met, the clone was excluded from GLMM and CNV RF analyses.

### Choice of the Method to Estimate CNV

Methods based on depth of sequencing (e.g., Abyzov et al. 2011) are popular to assess the extent of CNV along genomes. However, this kind of algorithm cannot be applied to capture data as the enrichment procedure increases the variance of sequencing depth among loci, making estimations unreliable. Therefore, we used the algorithm of Rigaill et al. (2012), especially designed for capture data. This method relies on the observation that, at constant sequencing effort, the depth of sequencing of any locus is consistent among individuals if their genomic structure is similar. Thus, the method performs pairwise comparisons of sequencing depth between tested individuals and a “gold standard” (e.g., a cancer cell sample vs. a healthy cell sample) for which we may know the CN for each target. The algorithm first defines chromosome sections that are homogeneous in terms of CN by inferring the positions of break points. For each chromosome section, a linear model is fitted to infer alpha, the ratio of CN between the gold standard and the tested individual (see fig. 1). Finally, different segmentation solutions are compared in a maximum-likelihood framework and the best one is retained (Rigaill et al. 2012). The method is currently implemented in the function “findOptimalSegmentations” of the R package “optimalCaptureSegmentation_0.9-4” available at http://bioinformatics.nki.nl/ocs/, last accessed September 24, 2014.

### Subtargets, PRbp, and Gold Standard

Targets were divided into “subtargets” of 50 bp in order to obtain good localizations of the CNV break points. The subdivision process was optimized using a homemade R script in order that the length of subtargets at the target edges was as close as possible of 50 bp. Because the PICARD tool module “CalculateHsMetrics” that we used to measure the sequencing depth of each subtarget could not obtain independent estimates of contiguous subtargets, we separated them by gaps of 1 bp. In this way any target of, say, 183 bp was systematically divided into four subtargets of 40, 50, 50, and 40 bp. For each individual, we calculated the PRbp for each subtarget (PRbp—the average sequencing depth per base pair divided by the total number of reads for this individual) as input for the algorithm. Following Rigaill et al. (2012), we square root transformed PRbp values in order to stabilize the variance among subtargets.

Because the pea aphid reference genome is from an American clone from *Medicago sativa* (LSR1), we used individuals from this race to define our gold standard in order to have it as similar as possible to the reference genome. As recommended by Rigaill et al. (2012), we defined the standard by averaging the PRbp over ten *Medicago* clones from the same genetic cluster in order to smooth the effect of rare CN variants. In order to deal with subtargets showing extensive CNV among these ten individuals, we removed subtargets with the lowest and highest 1% of PRbp values in the gold standard.

### Data Cleaning and Transformation

To obtain more reliable evaluation of CNV, we restrained our analysis to targets and subtargets present only once in Assembly 2.1 of the pea aphid genome (see Discussion and section 8 of Teo et al. 2012). We used GMAP (Wu and Watanabe 2005) with the default options to find the best match of each of our targets in the reference genome. Targets with more than one best match were discarded for subsequent analyses. Because low PHRED mapping scores may also reflect possible duplications in the reference genome (Stampy gives a score of 0 for reads mapping equally well in different locations of the genome), we also excluded subtargets with more than 5% of reads having a PHRED score lower than 10.

Although sequencing results were very consistent among individuals of the same libraries, individuals from different libraries showed heterogeneity in their enrichment patterns (supplementary fig. S1, Supplementary Material online), and thus in their relation to the gold standard (supplementary fig. S3, Supplementary Material online). In order to account for this experimental source of variance, we first defined our gold standard using *Medicago* individuals from different batches in order to conservatively include some experimental variance in the standard (supplementary fig. S3, Supplementary Material online). Second, we estimated for each individual the differences in enrichment patterns from the gold standard by fitting a third degree polynomial: y = ax^3 ^+ bx^2 ^+ cx where x was the PRbp of the standard and y the PRbp of the assessed individual. Finally, we corrected for the observed difference by transforming y using the inverse of the fitted polynomial (see supplementary fig. S3, Supplementary Material online). Note however that this transformation does not correct for other biases of unknown origin causing high variance across subtargets. Therefore, we had to remove all 12 clones of one lane (D0CM0ABXX_1) and three further clones that were still unsuitable for CNV estimation after transformation (supplementary table S4 and fig. S3, Supplementary Material online), reducing our sample to 104 clones (supplementary table S3, Supplementary Material online).

Because we kept only targets and subtargets with a single copy in the reference genome, we expected our *Medicago* gold standard to have one copy per chromosome for most subtargets (see Results). Therefore, in order to reduce the noise in the results even further, we rounded the CN estimations given by “findOptimalSegmentations” to the closest half (i.e., 0, 0.5, 1, 1.5, etc.).

### Response Variables Used in GLMM

We performed three GLMM to investigate variables influencing the dynamics of CNV between gene categories. In order to account for race-related effects, observations were made per gene in each race independently. Therefore, from our sample of 104 clones, 21 whose race membership was uncertain were excluded from CNV estimation (supplementary table S3, Supplementary Material online). Polymorphism was considered within races so that subtargets showing CNV among races but no variation within races were considered not variable (such subtargets were rare). We did not try to distinguish between duplication and deletion effects in these analyses due to lack of power. Response variables of the three GLMM were defined as follows for each gene and race: 1) CNV presence/absence—a gene was considered to show CNV in a race if at least one of its subtargets presented a CN variant (i.e., CN ≠ 1X) in at least one individual of this race, 2) completely duplicated or deleted (CDD) genes versus partially duplicated or deleted genes—a gene was considered CDD if all of its subtargets showed CN variants in at least one individual in the race, and 3) CNV frequency—the proportion of individuals in a race with CN ≠ 1X. In each GLMM, the relation between the response and explanatory variables was modeled using a logit function and a binomial error structure.

We restrained our analyses to *Or*, *Gr*, *P450,* and control genes as sample sizes of genes in other categories were too small to allow proper statistical analyses. Although they are biologically very interesting, we refrained from including promoters. We were concerned that they might not constitute a homogeneous locus category (in contrast to genes that are part of the same gene family or control genes that were randomly chosen to represent the genome). Also, their genetic structure would have made the analyses more complicated or even biased (small size associated with very low variance, no introns/exons).

### Explanatory Variables Used in GLMM

The following variables were modeled as fixed effects: 1) The gene CSL (natural logarithm), 2) the gene ISL (natural logarithm), 3) whether or not genes were truncated during in silico cleaning steps (see below), 4) the gene family, and 5) the genetic proximity to the *Medicago* gold standard. The influences of genes and races per se were also modeled as random effects. The binary variable “truncated gene” was included in order to account for potential biases introduced during the data cleaning step (see Data Cleaning and Transformation). By removing targets or subtargets present more than once in the genome, we shortened (truncated) some genes to a small fraction of their initial length (note that CSL and ISL were obtained from complete genes, i.e., lengths before truncation). Because they were shorter on average, a priori we expected these genes to have an artificially lower chance of showing CNV and a greater chance of appearing to be completely duplicated (i.e., a gene truncated to 20% of its initial length may appear completely duplicated in our analysis whereas it is not in reality). The effect of the gene family was tested using treatment contrasts, considering the control genes as the reference group (i.e., model intercept): Therefore differences between multigene families themselves were not tested directly. The genetic proximity to the gold standard was set as a binary variable (closely or not closely related) with the three races *Medicago*, *Pisum,* and *Trifolium* considered closely related. This dichotomy does not fully capture the continuum of divergence observed among races (Peccoud, Ollivier, et al. 2009; Ferrari et al. 2012), but it does simplify the analysis and separate strongly divergent races from the three races that were most closely related to the gold standard in our sample (results from our SNP data—not shown—and fig. 6).

### Model Description and GLMM Methodology

For our three GLMM, we used multimodel selection and model averaging techniques to assess the effects of explanatory variables, and their interactions, on CNV dynamics. Multimodel selection allows the estimation of the relative importance of each variable in a model. From a comprehensive model, a set of all the possible combinations of submodels were generated and these submodels were ranked according to their Akaike Information Criterion (AIC). Submodels with AIC not greater than the AIC of the best model plus a given threshold (Δ_i_) were kept as a set of best submodels. Model averaging was then used on this set to compute averaged parameter values and their associated probabilities (for more information, see Burnham and Anderson 2002; Grueber et al. 2011; Symonds and Moussalli 2011). Because we did not detect any strong influence of Δ_i_, we chose to keep it large with a value of 10. We tried to keep our comprehensive models as simple as possible in order to keep the set of submodels relatively small and to simplify the inferences. Therefore, we sequentially ran each GLMM and removed variables that were significant in none of the submodels (as well as interactions involving these variables) with the sole exceptions of the terms “gene family” and “truncated” that we tested in each GLMM. All the analyses were conducted using R 3.1.0 and the packages “lme4_1.0-5” (Bates et al. 2013) and “MuMIn_1.9.13” (Bartoń 2013).

### Procedure to Detect Genes Best Discriminating Host Races

We used V_ST_, an analog of F_ST_ for CNV (Redon et al. 2006), in order to describe the extent of CNV among races. Briefly, V_ST_ is calculated as (V_T_ − V_S_)/V_T_, where V_T_ is the variance of CN observed across all individuals and V_S_ is the within-race variance of CN, averaged across races. Unlike Redon et al. (2006), we used our rounded CN values rather than a log2 transformed CN but this difference did not qualitatively alter the results. We averaged V_T_ and V_S_ over segments within a gene that had distinct CN. We then used RF to assess the relative importance of variables (i.e., CN for each subtarget) for classification of clones into host races. Host race genetic clusters were defined a priori (“supervised” classification RF) based on results from the SNP analysis (83 clones with clear race membership assignment as “training set” and the other 21 clones as “test set”; supplementary table S3, Supplementary Material online). To avoid the problem of nonindependence of subtargets that are physically linked, we adopted a recursive procedure in four rounds to retain only independent subtargets representative of genes and promoters. A first RF of 10,000 trees was performed using all polymorphic subtargets (i.e., a matrix of 83 clones and 2,041 subtargets). This showed that 5,000 trees were actually enough to ensure the stability of the variable importance estimates made by the RF for our data set (not shown). A second round was then run by keeping only the most informative subtarget for each target. Finally, the third and fourth rounds were conducted similarly but by keeping the most informative subtarget per gene then scaffold, respectively. Note that after each round, all noninformative subtargets (i.e., with a mean Gini Importance of 0) were removed from subsequent analyses and that promoters were considered as genes in this procedure.

### Tests of Nonrandom Distribution of Most Discriminating Subtargets

In order to properly test characteristics of the best discriminating subtargets (overrepresentation of gene families or CDD genes), we performed nonparametric tests on empirical null distributions. It was necessary to formulate null hypotheses respecting the same complex sampling procedure as described above. We therefore generated a set of 5,000 random samples from which all tests were derived. These random samples were produced in four steps. First, a set of loci were randomly drawn but with the constraint of simultaneously matching two features observed in the real data during round 3 of the above procedure: 1) The number of genes and promoters retained and 2) the gene–gene, promoter–promoter, and gene–promoter probabilities of being on the same scaffold. Second, loci were ranked randomly without distinction between genes and promoters. Third, for each scaffold, only the subtarget with the best rank was kept. Forth, of the remaining subtargets those with the lowest ranks were discarded in order to match the number of subtargets observed in round 4 of the above procedure (i.e., 114 subtargets). Miscellaneous empirical null distributions (number of *Gr* in the top 40, sum of the rank of *Or* in the top 40, sum of the rank of CDD genes, etc.) were then derived from these 5,000 random samples in order to perform one-tailed statistical tests.

## Supplementary Material

Supplementary figures S1–S3 and tables S1–S6 are available at *Molecular Biology and Evolution* online (http://www.mbe.oxfordjournals.org/).

Supplementary Data

## Data and Code Access

Sequencing reads sorted by individuals (Edinburgh Genomics tags) have been deposited in the EBI Sequence Read Archive (SRA) with project accession no. PRJEB6325 (individual accession numbers are given in supplementary table S4, Supplementary Material online). Some intermediate data sets and result files needed to reproduce parts of the results outside our pipeline (e.g., matrix of raw PRbp counts, matrix of raw alpha from the “optimalCaptureSegmentation” analysis, R data frames used in GLMM, NJ tree showing data per capture pools and sequencing lanes, extended fig. 1 including all targeted loci) can be found on DRYAD, doi:10.5061/dryad.jf29v.

The analytical framework set up to analyze CNV distribution (including CN estimation from PRbp, GLMM, RF analyses and general statistics) is publicly available on a git repository https://github.com/lduvaux/Duvaux_et_al_2014, last accessed September 24, 2014 and reusable under the conditions of the General Public License (v3). A copy of the project has been deposited at https://zenodo.org/ (last accessed September 24, 2014) under the DOI:10.5281/zenodo.11857. Other miscellaneous scripts and command lines used to process sequencing results can be made available by the authors on request.
