# Supplementary material for: Dynamics of Copy Number Variation in Host Races of the Pea Aphid
Source: Mol Biol Evol. 2014 Sep 18;32(1):63–80. doi: 10.1093/molbev/msu266 (PMC4271520; doi:10.1093/molbev/msu266)
Supplement: Supplementary Data [file supp_32_1_63__index.html]

Dynamics of copy number variation in host races of the pea aphid — Dynamics of Copy Number Variation in Host Races of the Pea Aphid — Dynamics of Copy Number Variation in Host Races of the Pea Aphid — Supplementary Data 

# Dynamics of Copy Number Variation in Host Races of the Pea Aphid

## Supplementary Data

files

**Files in this Data Supplement:**

- Supplementary Data - pdf file
- Supplementary Data - pdf file
- Supplementary Data - jpg file
- Supplementary Data - docx file
- Supplementary Data - xlsx file
