# Supplementary material for: Dynamics of Copy Number Variation in Host Races of the Pea Aphid
Source: Mol Biol Evol. 2014 Sep 18;32(1):63–80. doi: 10.1093/molbev/msu266 (PMC4271520; doi:10.1093/molbev/msu266)
Supplement: Supplementary Data [file supp_msu266_Duvaux_CNV-PeaAphid_FigS1_Sequencing_stats.pdf]

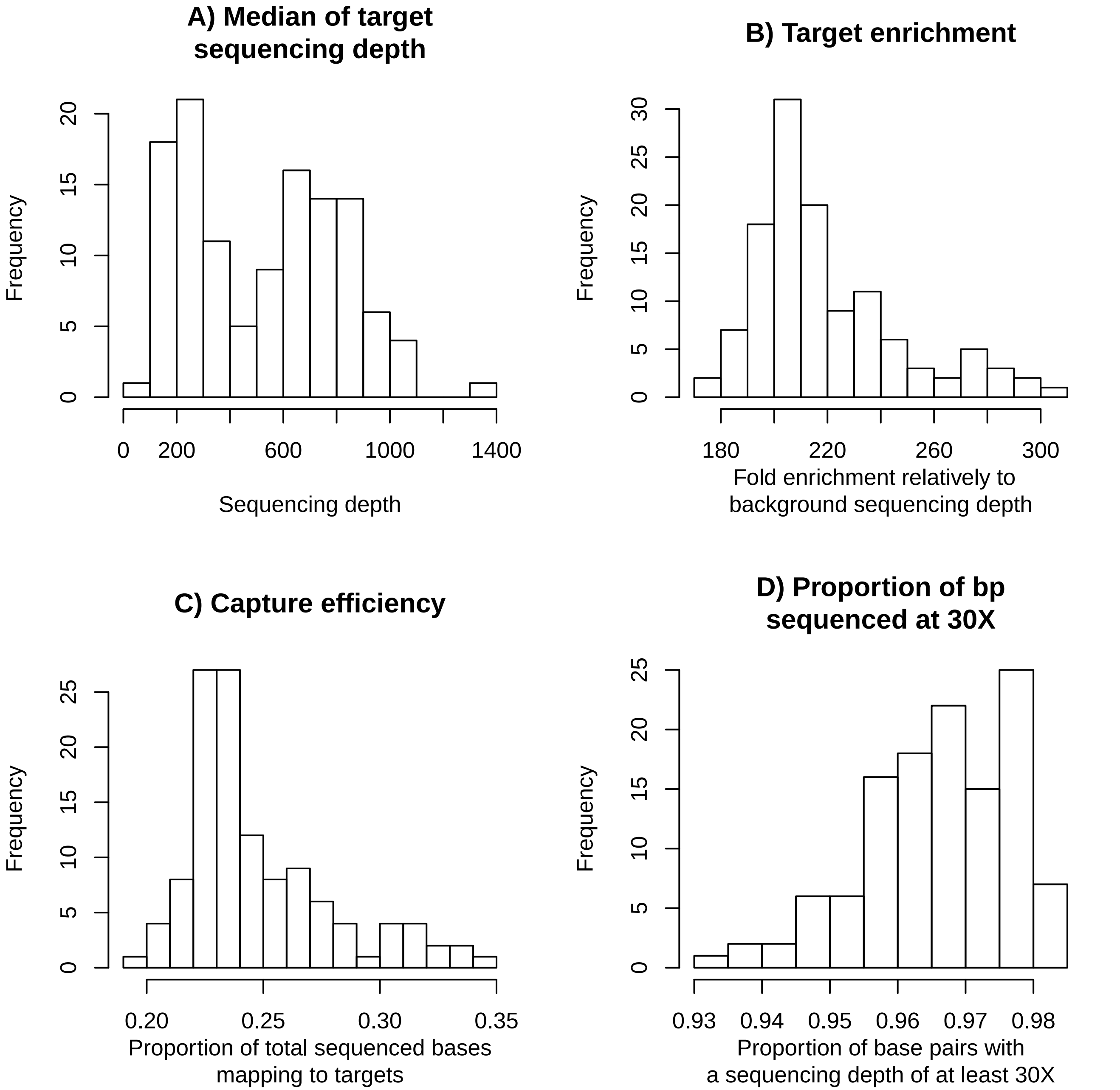
A) Median of target sequencing depth
Frequency
0
5
10
15
20
0
200
600
1000
1400
Sequencing depth
B) Target enrichment
Frequency
0
5
10
15
20
25
30
180
220
260
300
Fold enrichment relatively to background sequencing depth
C) Capture efficiency
Frequency
0
5
10
15
20
25
0.20
0.25
0.30
0.35
Proportion of total sequenced bases mapping to targets
D) Proportion of bp sequenced at 30X
Frequency
0
5
10
15
20
25
0.93
0.94
0.95
0.96
0.97
0.98
Proportion of base pairs with a sequencing depth of at least 30X
