# Supplementary material for: Dynamics of Copy Number Variation in Host Races of the Pea Aphid
Source: Mol Biol Evol. 2014 Sep 18;32(1):63–80. doi: 10.1093/molbev/msu266 (PMC4271520; doi:10.1093/molbev/msu266)
Supplement: Supplementary Data [file supp_msu266_Duvaux_CNV-PeaAphid_FigS3_ResultsDataTransformation.pdf]

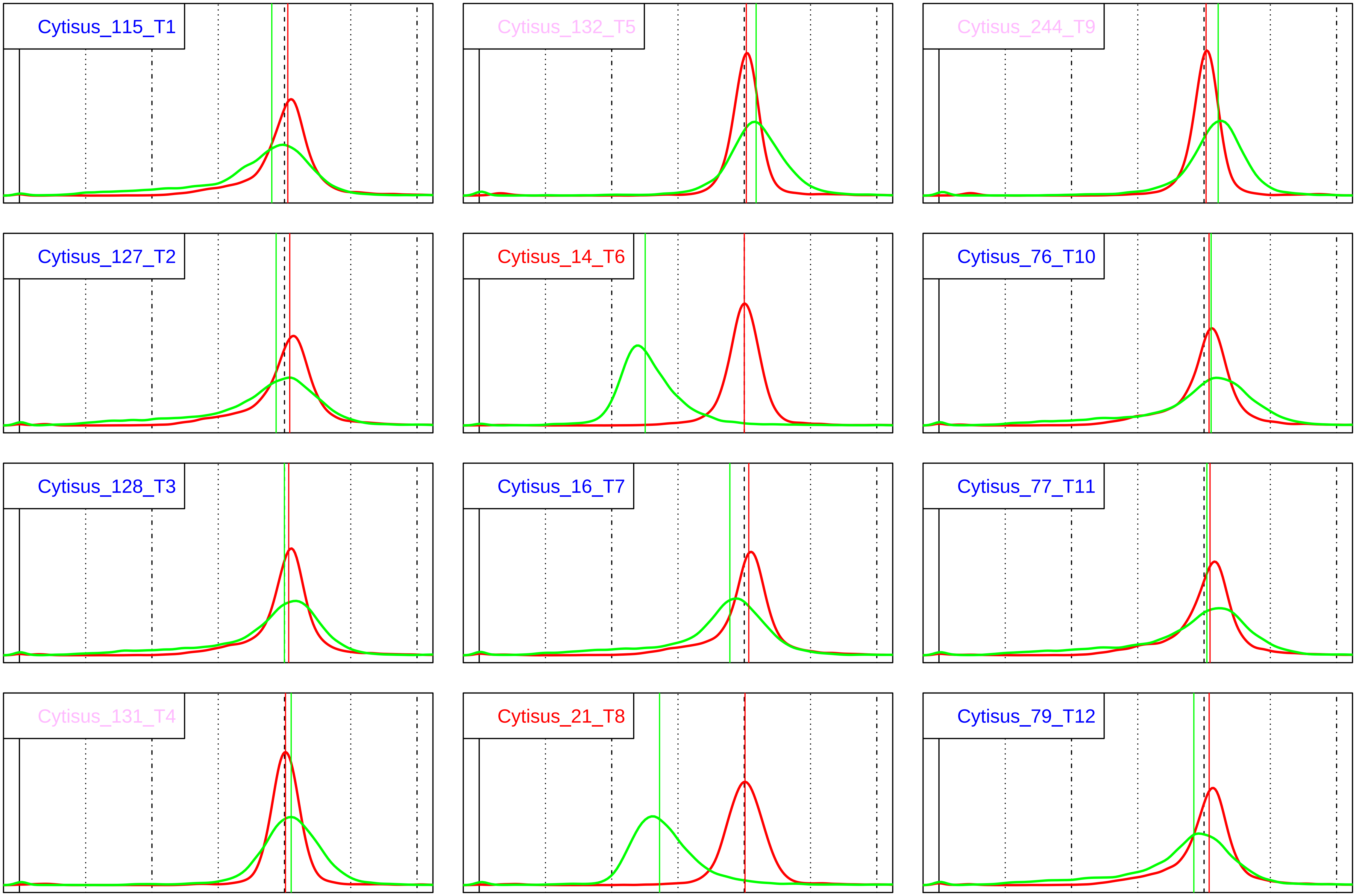
Cytisus_115_T1
Cytisus_132_T5
Cytisus_244_T9
Cytisus_127_T2
Cytisus_14_T6
Cytisus_76_T10
Cytisus_128_T3
Cytisus_16_T7
Cytisus_77_T11
Cytisus_131_T4
Cytisus_21_T8
Cytisus_79_T12

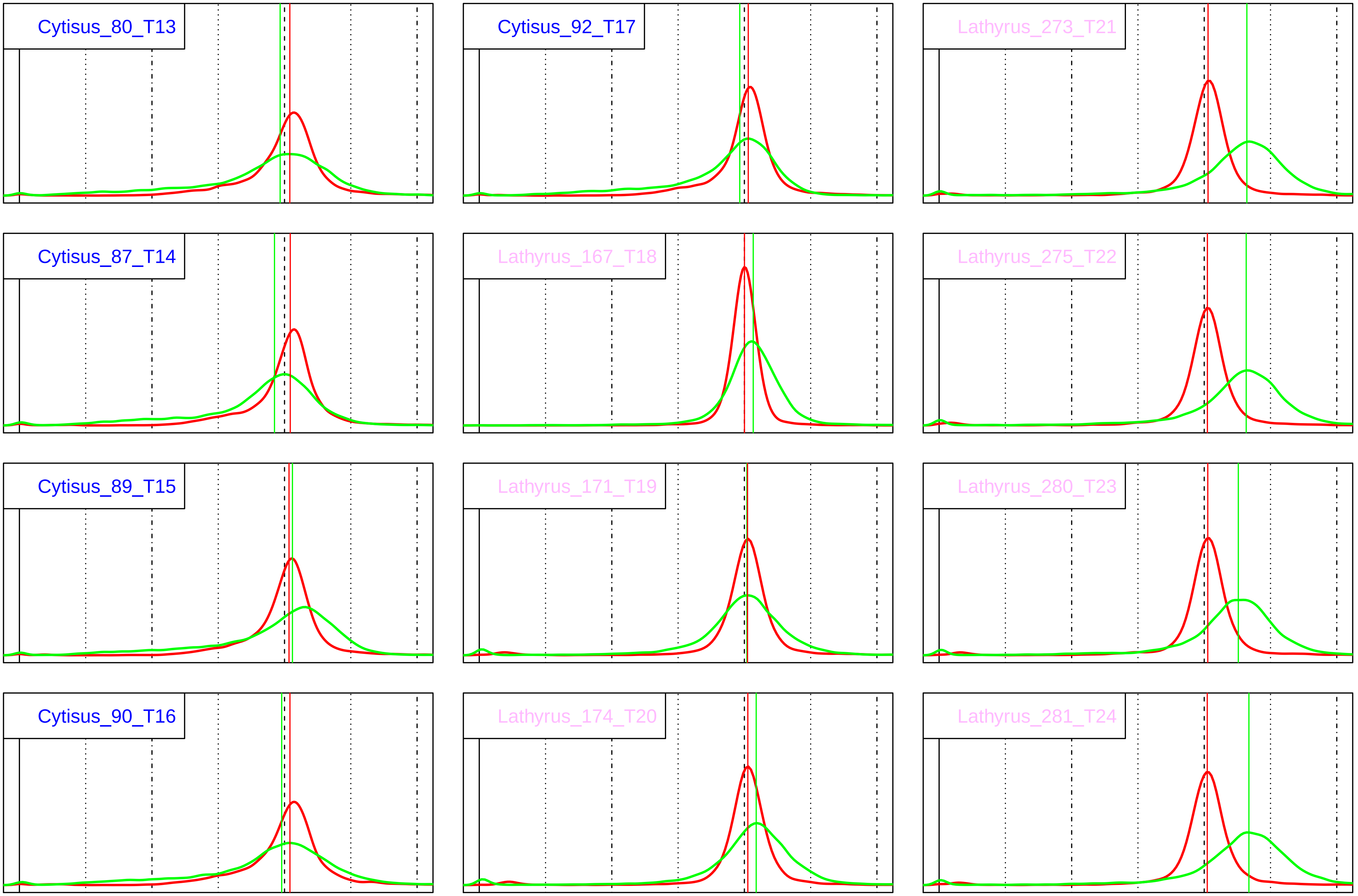

Cytisus_80_T13
Cytisus_92_T17
Lathyrus_273_T21
Cytisus_87_T14
Lathyrus_167_T18
Lathyrus_275_T22
Cytisus_89_T15
Lathyrus_171_T19
Lathyrus_280_T23
Cytisus_90_T16
Lathyrus_174_T20
Lathyrus_281_T24

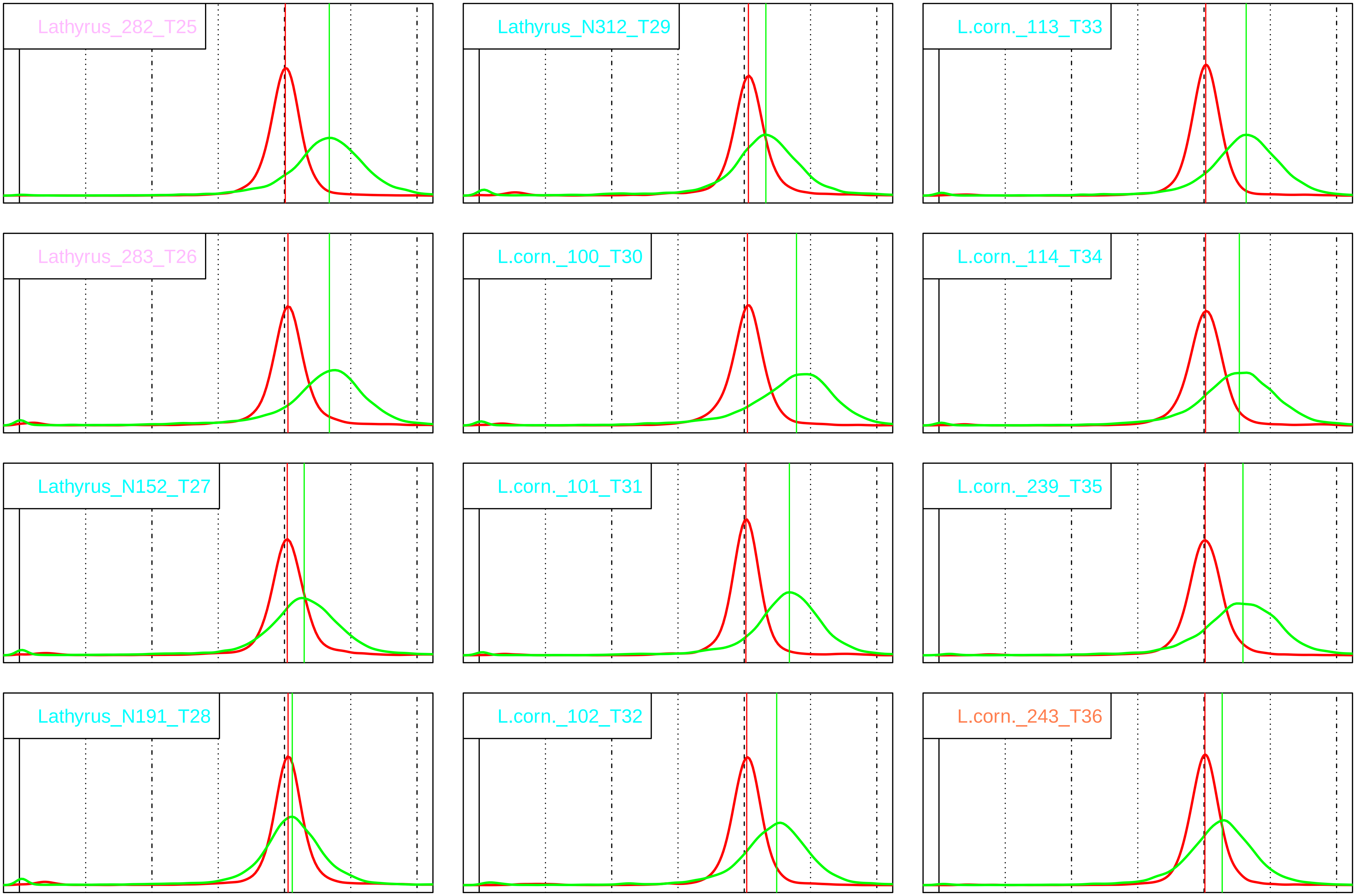
Lathyrus_282_T25
Lathyrus_N312_T29
L.corn._113_T33
Lathyrus_283_T26
L.corn._100_T30
L.corn._114_T34
Lathyrus_N152_T27
L.corn._101_T31
L.corn._239_T35
Lathyrus_N191_T28
L.corn._102_T32
L.corn._243_T36

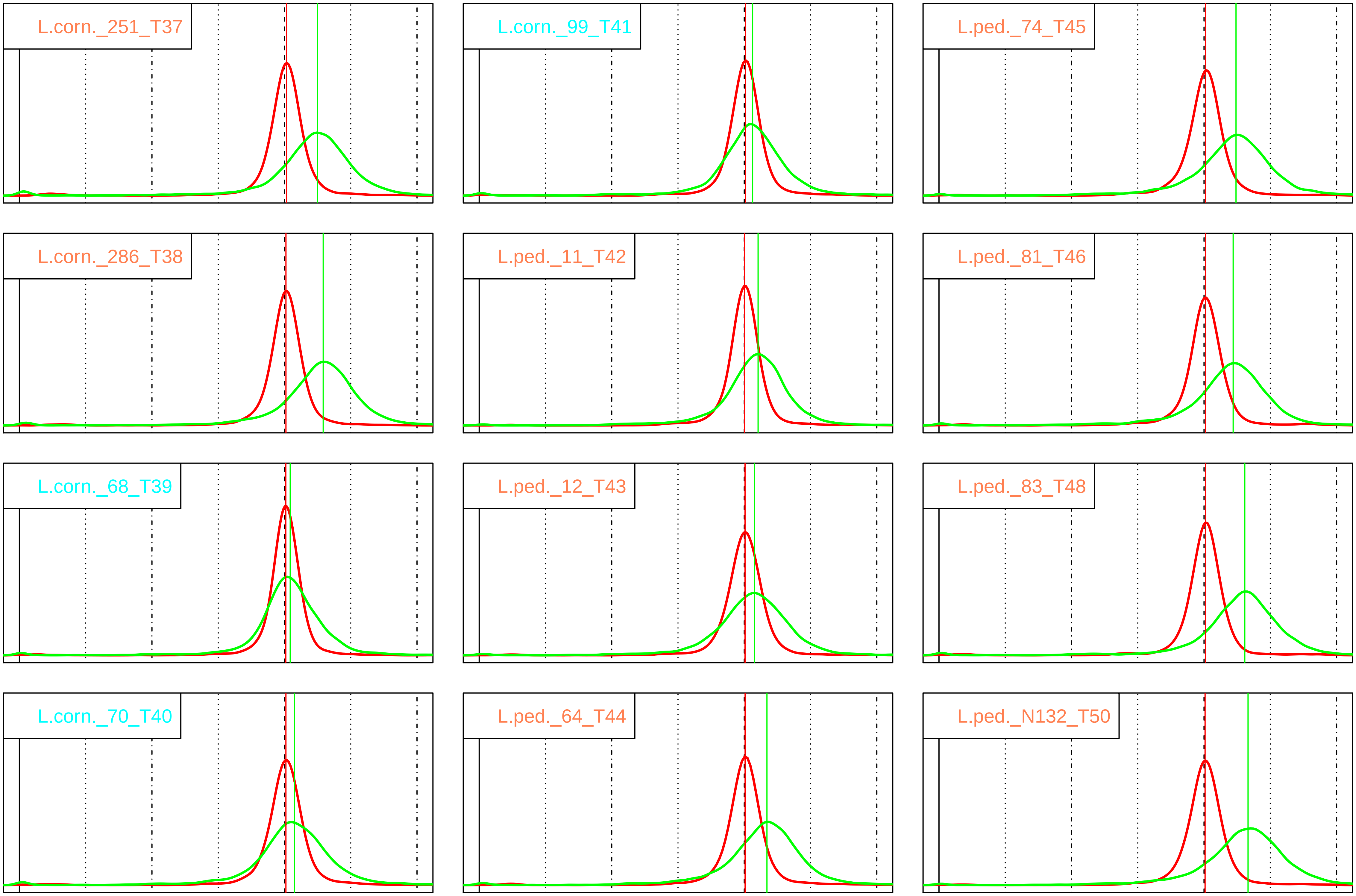

L.corn._251_T37
L.corn._99_T41
L.ped._74_T45
L.corn._286_T38
L.ped._11_T42
L.ped._81_T46
L.corn._68_T39
L.ped._12_T43
L.ped._83_T48
L.corn._70_T40
L.ped._64_T44
L.ped._N132_T50

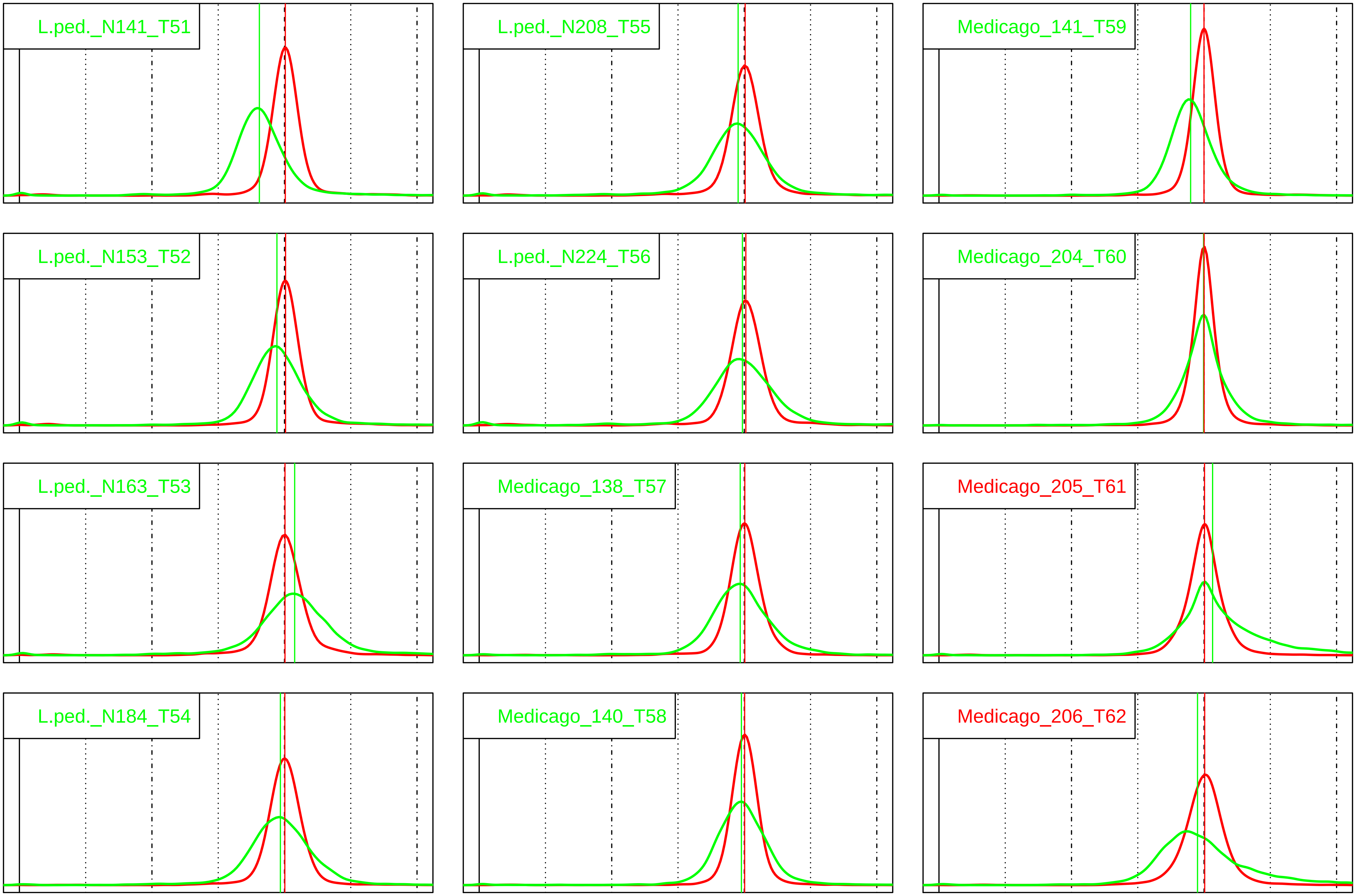
L.ped._N141_T51
L.ped._N208_T55
Medicago_141_T59
L.ped._N153_T52
L.ped._N224_T56
Medicago_204_T60
L.ped._N163_T53
Medicago_138_T57
Medicago_205_T61
L.ped._N184_T54
Medicago_140_T58
Medicago_206_T62

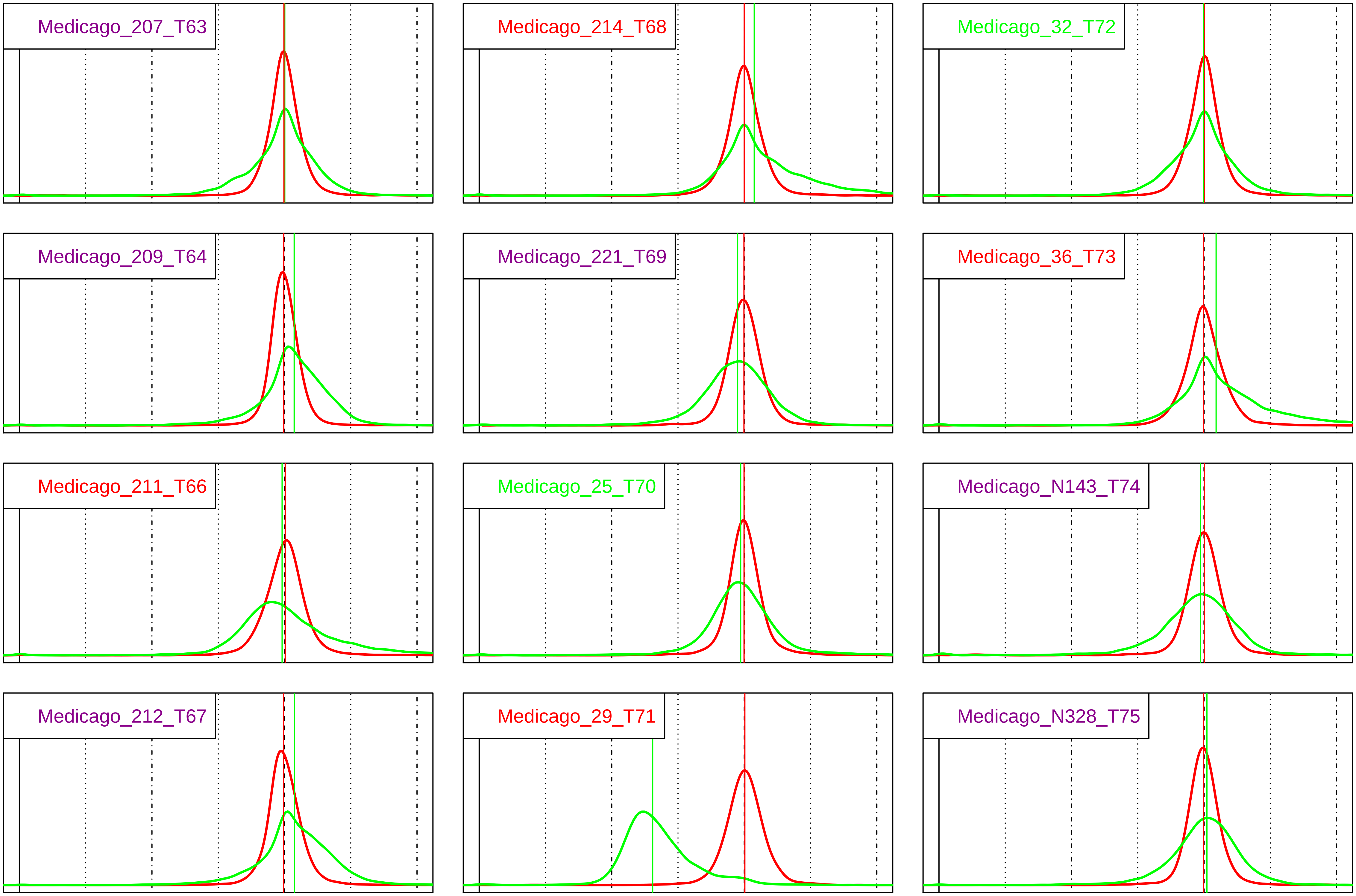

Medicago_207_T63
Medicago_214_T68
Medicago_32_T72
Medicago_209_T64
Medicago_221_T69
Medicago_36_T73
Medicago_211_T66
Medicago_25_T70
Medicago_N143_T74
Medicago_212_T67
Medicago_29_T71
Medicago_N328_T75

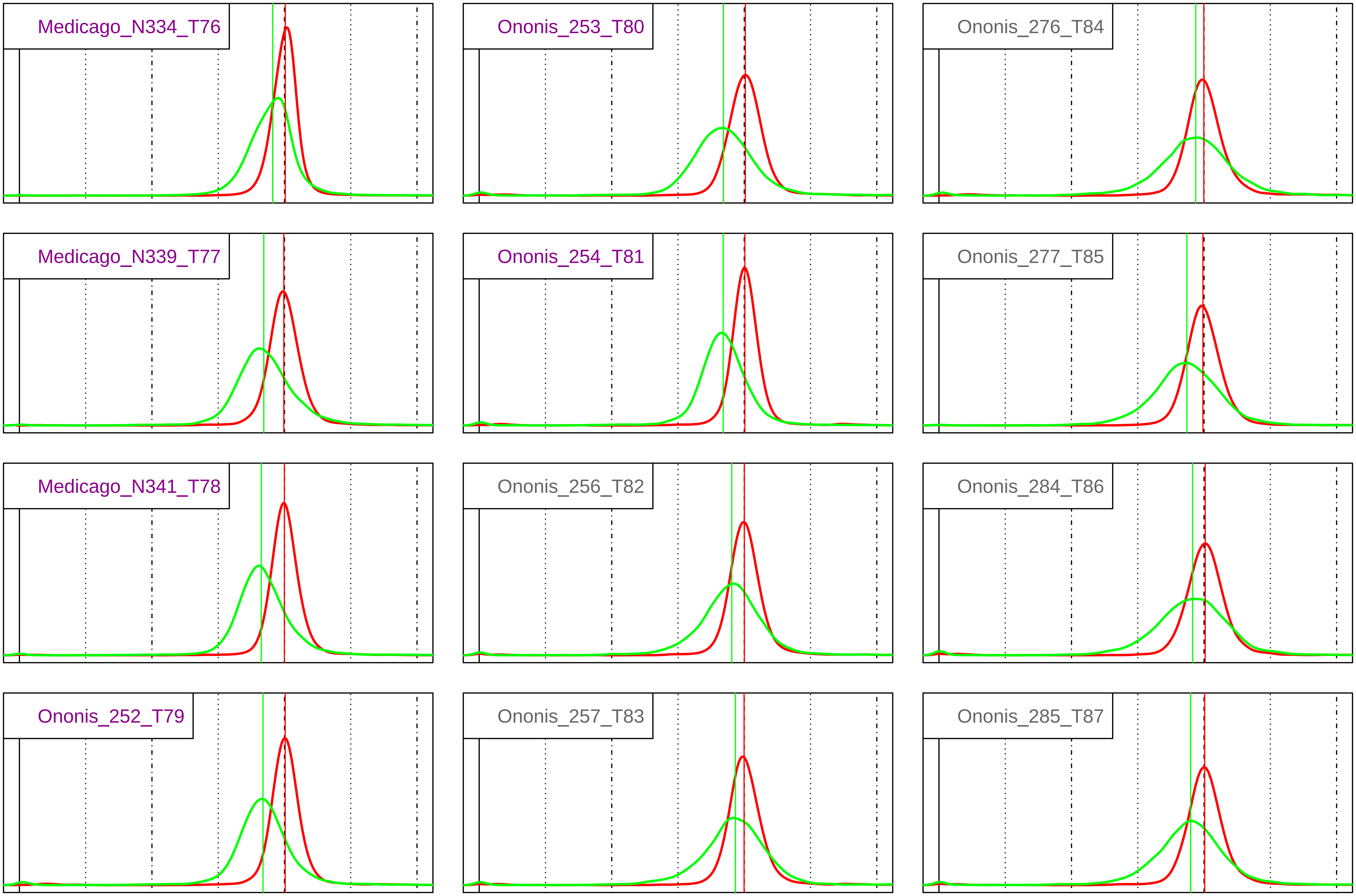
Medicago_N334_T76
Ononis_253_T80
Ononis_276_T84
Medicago_N339_T77
Ononis_254_T81
Ononis_277_T85
Medicago_N341_T78
Ononis_256_T82
Ononis_284_T86
Ononis_252_T79
Ononis_257_T83
Ononis_285_T87

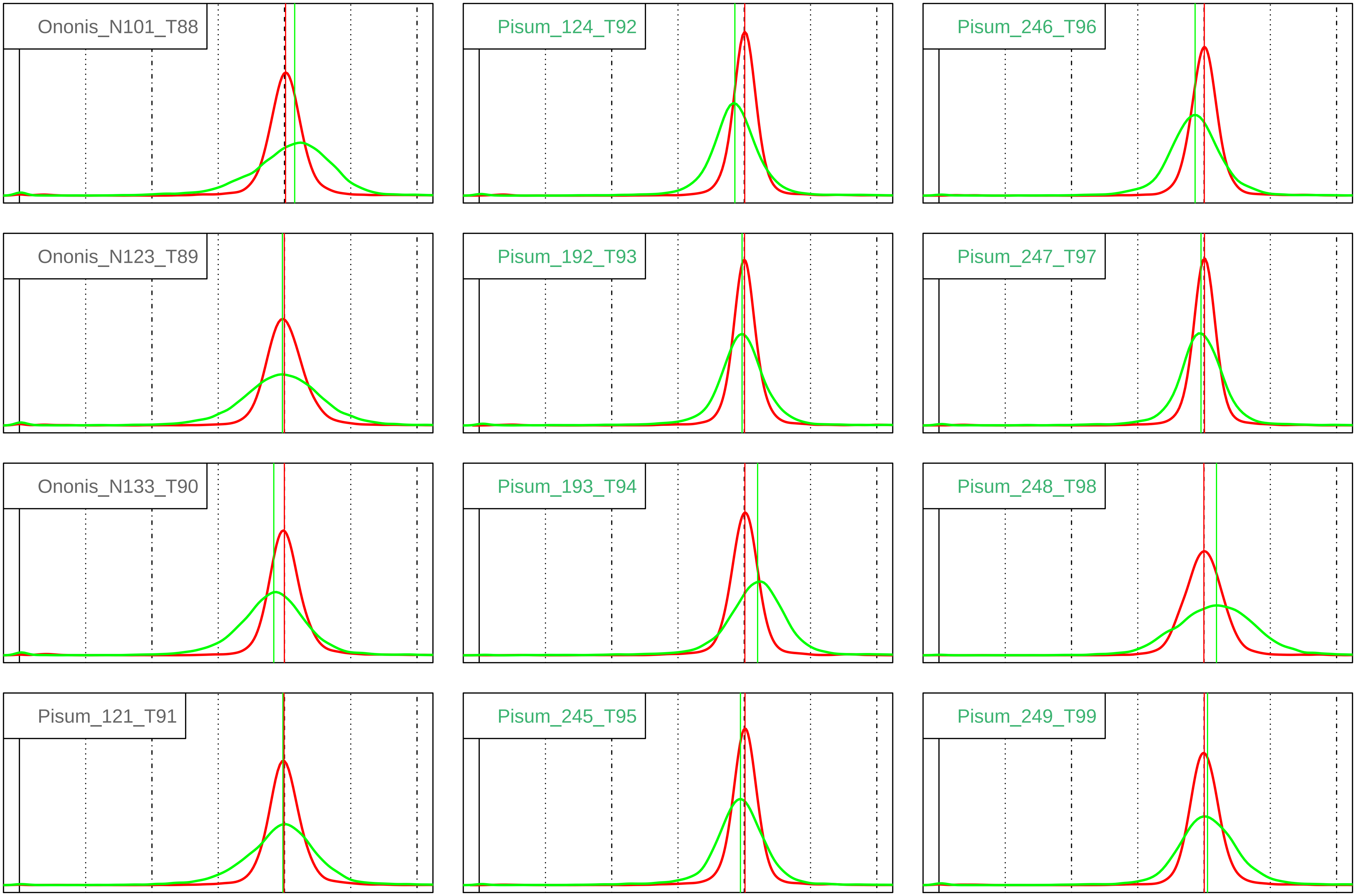
Ononis_N101_T88
Pisum_124_T92
Pisum_246_T96
Ononis_N123_T89
Pisum_192_T93
Pisum_247_T97
Ononis_N133_T90
Pisum_193_T94
Pisum_248_T98
Pisum_121_T91
Pisum_245_T95
Pisum_249_T99

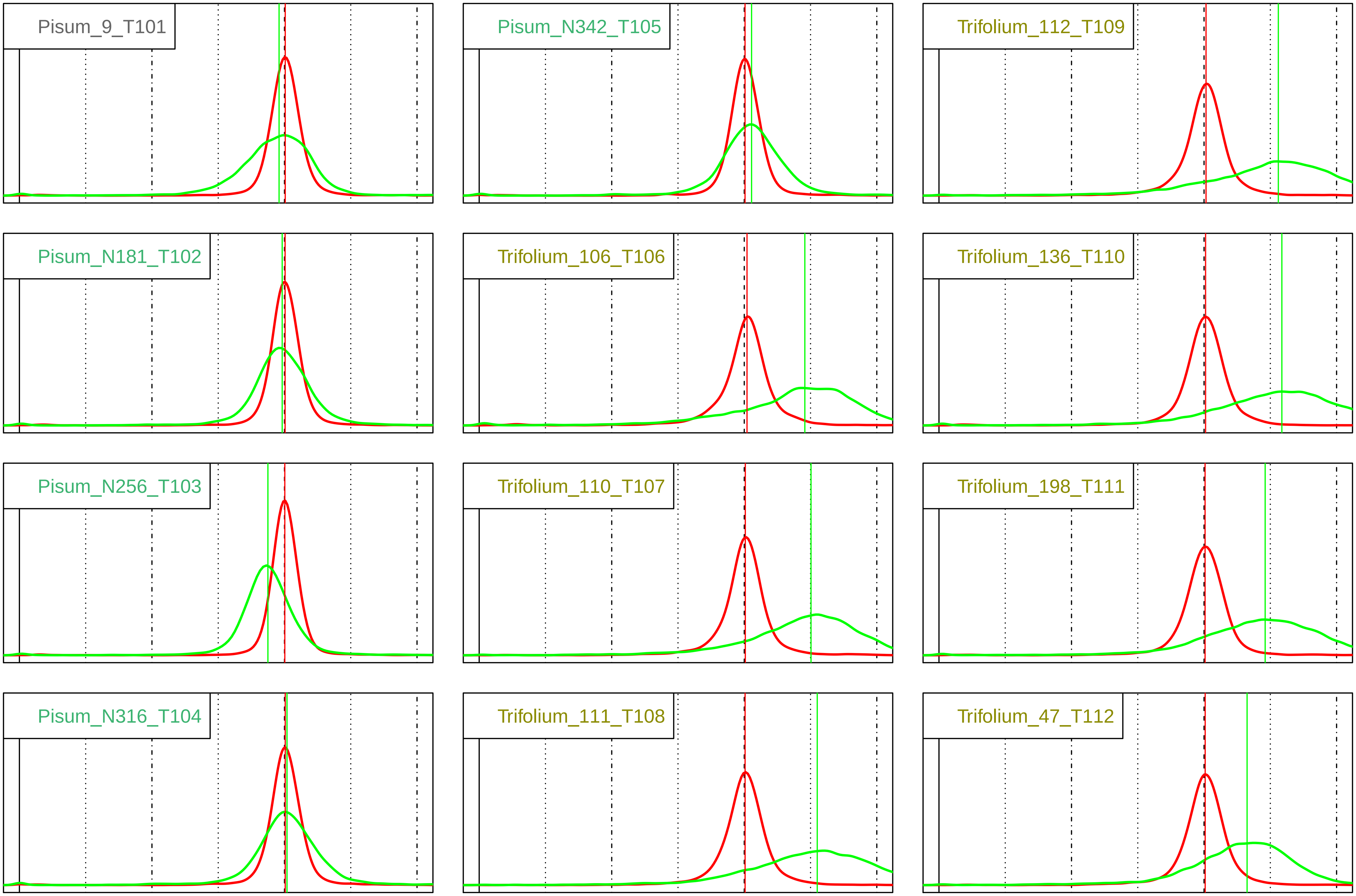
Pisum_9_T101
Pisum_N342_T105
Trifolium_112_T109
Pisum_N181_T102
Trifolium_106_T106
Trifolium_136_T110
Pisum_N256_T103
Trifolium_110_T107
Trifolium_198_T111
Pisum_N316_T104
Trifolium_111_T108
Trifolium_47_T112

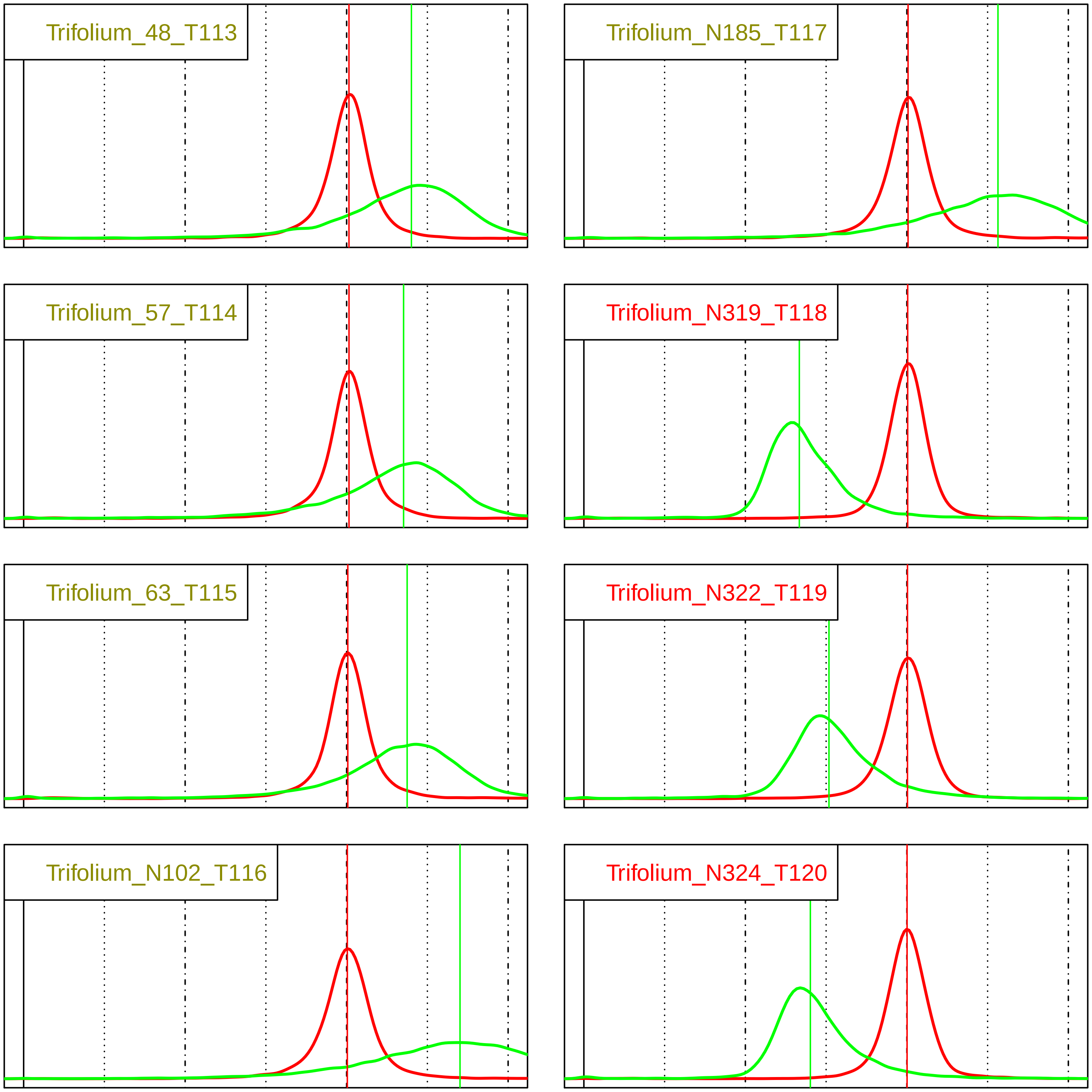
Trifolium_48_T113
Trifolium_N185_T117
Trifolium_57_T114
Trifolium_N319_T118
Trifolium_63_T115
Trifolium_N322_T119
Trifolium_N102_T116
Trifolium_N324_T120
