# Supplementary figures and images for: Dynamics of Copy Number Variation in Host Races of the Pea Aphid
Source: Mol Biol Evol. 2014 Sep 18;32(1):63–80. doi: 10.1093/molbev/msu266 (PMC4271520; doi:10.1093/molbev/msu266)

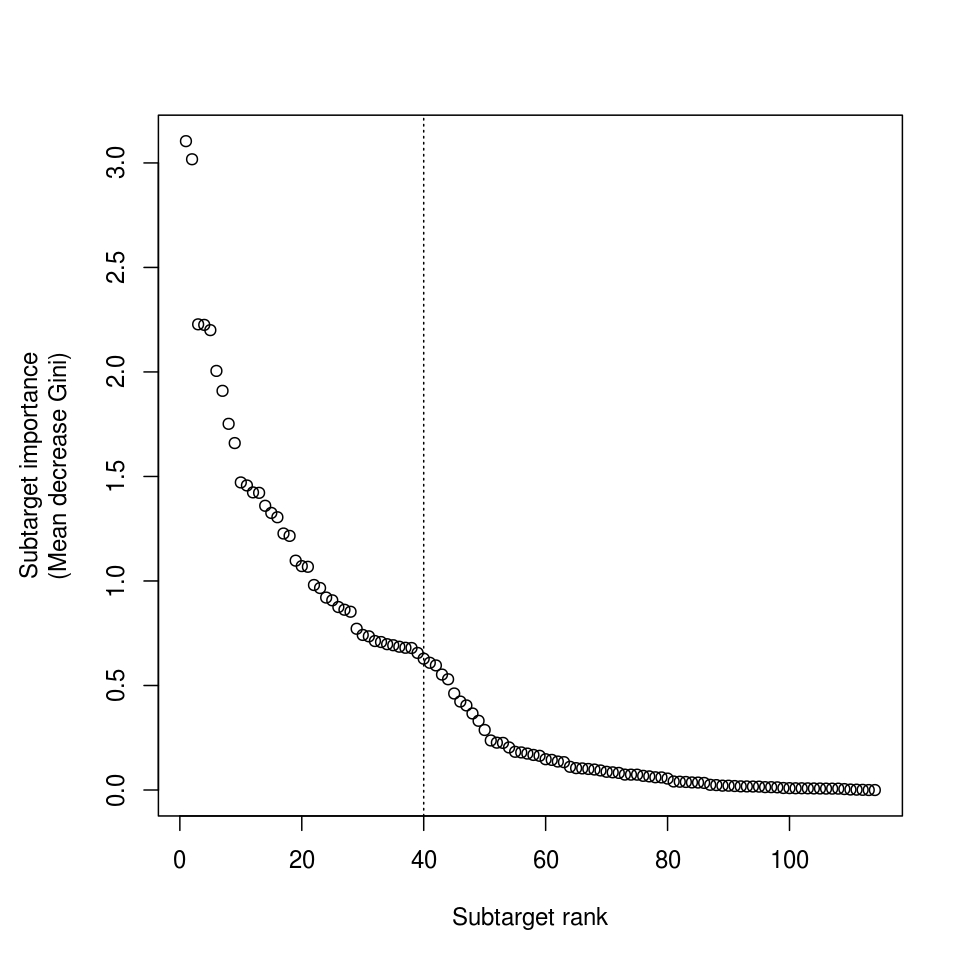

Supplement: Supplementary Data [file supp_msu266_Duvaux_CNV-PeaAphid_FigS2_MeanDevcreaseGini.jpg]
