# Supplementary material for: Dynamics of Copy Number Variation in Host Races of the Pea Aphid
Source: Mol Biol Evol. 2014 Sep 18;32(1):63–80. doi: 10.1093/molbev/msu266 (PMC4271520; doi:10.1093/molbev/msu266)
Supplement: Supplementary Data [file supp_msu266_Duvaux_CNV-PeaAphid_Sup.Fig_Legends.docx]

Figure S1. Distributions of four capture metrics by aphid clone. The last three metrics were derived from PICARD tools “HsMetrics”; for definitions see http://picard.sourceforge.net/picard-metric-definitions.shtml#HsMetrics. A) Median of target sequencing depth. B) Target enrichment (PICARD metric “FOLD_ENRICHMENT”). C) Capture efficiency (PICARD metric “ON_BAIT_BASES”/“PF_UQ_BASES_ALIGNED”). D) Proportion of base pairs sequenced at 30X at least (PICARD metric "PCT_TARGET_BASES_30X").

Figure S2. Importance of the most informative subtargets for discrimination among races. Subtargets are sorted by rank from the most to the least important. The vertical dotted line indicates the top 40 (see main text).

Figure S3. Distributions of the ratio of PRbp between the gold standard and samples of interest. The colours of the sample names correspond to different sequencing lanes. The green and red curves represent the data before and after the polynomial transformation, respectively (the coloured vertical lines denote the distribution medians). Vertical black lines: the plain, the dotted, the dashed and the dot-dashed lines represent values 0, [0.25, 0.75, 1.25], 1 and [0.5, 1.5], respectively. For samples of the lane D0CM0ABXX_1 (dark blue, e.g. Cytisus_115_T1), note the leftward tails present in both green and red distributions revealing a very strong influence of the library preparation. This anomaly was responsible for too great a number of subtargets having CN<1. The relevant samples were therefore discarded. For clarity, all the distributions have been truncated at 1.5. PRbp: proportion of reads per base pair, CN: copy number.
